# Division and Regrowth of Phase‐Separated Giant Unilamellar Vesicles**

**DOI:** 10.1002/anie.202014174

**Published:** 2021-03-24

**Authors:** Yannik Dreher, Kevin Jahnke, Elizaveta Bobkova, Joachim P. Spatz, Kerstin Göpfrich

**Affiliations:** ^1^ Biophysical Engineering Group Max Planck Institute for Medical Research Jahnstraße 29 69120 Heidelberg Germany; ^2^ Department of Physics and Astronomy Heidelberg University 69120 Heidelberg Germany; ^3^ Department of Cellular Biophysics Max Planck Institute for Medical Research Jahnstraße 29 69120 Heidelberg Germany; ^4^ Institute for Molecular Systems Engineering (IMSE) Heidelberg University Im Neuenheimer Feld 225 69120 Heidelberg Germany; ^5^ Max Planck School Matter to Life Jahnstraße 29 69120 Heidelberg Germany

**Keywords:** DNA structures, GUV division, osmosis, synthetic biology, vesicles

## Abstract

Success in the bottom‐up assembly of synthetic cells will depend on strategies for the division of protocellular compartments. Here, we describe the controlled division of phase‐separated giant unilamellar lipid vesicles (GUVs). We derive an analytical model based on the vesicle geometry, which makes four quantitative predictions that we verify experimentally. We find that the osmolarity ratio required for division is $\sqrt{2}$ , independent of the GUV size, while asymmetric division happens at lower osmolarity ratios. Remarkably, we show that a suitable osmolarity change can be triggered by water evaporation, enzymatic decomposition of sucrose or light‐triggered uncaging of CMNB‐fluorescein. The latter provides full spatiotemporal control, such that a target GUV undergoes division whereas the surrounding GUVs remain unaffected. Finally, we grow phase‐separated vesicles from single‐phased vesicles by targeted fusion of the opposite lipid type with programmable DNA tags to enable subsequent division cycles.

## Graphical abstract

We demonstrate division of phase‐separated lipid vesicles following quantitative predictions from an analytical model. The division is controlled by metabolic decomposition or light‐triggered uncaging of a fluorophore. This provides spatiotemporal control over the division of a target vesicle. We regrow phase‐separated vesicles by targeted fusion using DNA nanotechnology.

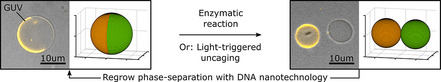

## Introduction

*“Omni cellulae e cellulae.”* From the point of view of modern science, Raspail's realization from 1825,^[1]^ popularized by Virchow,^[2]^ may state the obvious: Every living cell found on Earth today originates from a preexisting living cell. Bottom‐up synthetic biology, however, is challenging this paradigm with the vision to create a synthetic cell from scratch.[^3^, ^4^] Success unquestionably entails that the synthetic cells must have the capacity to produce offspring, making the implementation of synthetic cell division an exciting goal.[^5^, ^6^, ^7^, ^8^] Over the course of evolution, living cells have developed a sophisticated machinery to divide their compartments in a highly regulated manner. The reconstitution of a minimal set of cellular components seems to be a plausible albeit challenging route towards synthetic cell division.[^9^, ^10^, ^11^] These challenges leave room for creative approaches, seeking solutions beyond the mimicry of today's biological cells. One exciting strategy is to assemble a division machinery de novo, by designing active, not necessarily protein‐based nanomachines. DNA origami structures have been used to shape and remodel lipid vesicles,[^12^, ^13^, ^14^] although active force‐generating motors remain a distant goal. A shortcut towards synthetic cell division is the non‐autonomous mechanical division of liposomes,^[15]^ which may jump‐start exciting directions. The exploitation of physicochemical mechanisms, on the other hand, could lead to autonomous division. Noteworthy theoretical work describes the shape transformations of single‐phase[^16^, ^17^, ^18^] as well as phase‐separated liposomes[^19^, ^20^, ^21^] depending on the surface‐to‐volume ratio. Two vesicles connected with a tight neck have been theoretically predicted^[20]^ and can readily be observed in experiments. A remarkable recent report triggered shape transformations of lipid vesicles by an internal enzymatic reaction, but neck fission did not occur.^[22]^ There are few experimental reports describing the complete dissociation of small buds from a parent vesicle.[^23^, ^24^] Division into more equally sized compartments has once been reported as an occasional observation^[25]^ or it relied on multilamellar vesicles^[26]^ or liquid–liquid phase separation.^[27]^ Moreover, multilamellar fatty acid vesicle systems have been shown to deform and sometimes divide^[28]^ and recently, division was shown as a result of spontaneous curvature.^[29]^ However, we are still missing a well‐controlled division mechanism where designated vesicles divide with a success rate close to 100 %, combined with a suitable growth mechanism. This would be an important step for the field of bottom‐up synthetic biology since it could provide the basis for the evolution of synthetic cells.

Here, we experimentally demonstrate full spatiotemporal control over the division of phase‐separated GUVs with an unprecedented success rate. To predict the process quantitatively, we show that it is sufficient to look at the vesicle geometry. We describe the shape transformations of phase‐separated vesicles without fitting parameters, while previous theoretical work relies on membrane‐specific parameters.[^19^, ^20^, ^21^] From these geometrical considerations, we can extract the precise conditions required for division and thereby provide a reproducible and highly controlled division mechanism. Notably, we demonstrate that the division of GUVs can be regulated by a metabolic reaction or triggered locally by light. We further implement vesicle fusion via programmable DNA tags as a mechanism to regrow phase‐separated vesicles from single‐phased ones to enable subsequent division cycles. While our synthetic division mechanism distinctively differs from that of nowadays living cells, our results prompt to ask whether similar mechanisms may have sustained cell division at the onset of life[^30^, ^31^] or if remnants thereof may still play a role for the generation of intracellular vesicles or to support certain division processes of today's cells.[^32^, ^33^, ^34^]

## Results and Discussion

### Division of Phase‐Separated GUVs Triggered by Metabolic Decomposition

**Figure 1:**
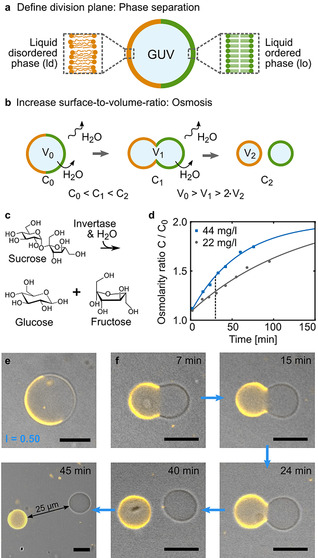
Division of phase‐separated GUVs. Schematic illustration of the division mechanism relying on a) phase separation of the GUVs and b) osmosis. C_0_, C_1_, and C_2_ denote the osmolarity outside of the GUVs and *V_1_*, *V_2_*, and *V_3_* describe their volume at different time points. c) Chemical reaction pathway of sucrose degradation catalyzed by the enzyme invertase. d) Osmolarity ratio *C*/*C*_0_ over time for GUV‐containing solutions composed of 300 mm sucrose, 10 mm HEPES (pH 7.4) and 44 mg L^−1^ (blue) or 22 mg L^−1^ invertase (gray). Error bars are too small to be visible. The data was fitted with limited growth fits (solid lines). The dotted black line indicates the time point at which division occurs (see f). e) Overlay of brightfield and confocal image of a phase‐separated GUV with equally large hemispheres (Lipid Mix 1, Table S2, ld phase labeled with LissRhod PE (orange), *λ*_ex_=561 nm). f) Confocal fluorescence time series depicting the division process in the presence of 44 mg L^−1^ invertase. The vesicles are fully separated and quickly diffuse apart after division (see 45 min time point). Scale bars: 10 μm.

GUVs—that is, micron‐sized vesicles enclosed by a single lipid bilayer—are the most commonly used compartment type for the assembly of synthetic cells.^[4]^ To realize a controllable and efficient mechanism for their division, we propose a strategy that is based on three steps: Step 1) Define the plane of division; Step 2) Increase the surface‐to‐volume ratio, and Step 3) Enable neck fission to allow for the formation of two smaller second‐generation compartments from a single large compartment. To realize Step 1, we choose lipid phase separation to define the plane of division as the interface of the liquid‐disordered (ld, orange) and the liquid‐ordered (lo, green) phase as illustrated in Figure 1 a. Hence, an increase in the surface‐to‐volume ratio (Step 2) requires a reduction of the GUV's inner volume. To this end, we exploit osmosis. An increase of the osmolarity outside the GUVs, that is, a higher concentration of solutes in the outer aqueous solution, causes water efflux through the GUV membrane^[35]^ as illustrated in Figure 1 b. Note that the number of lipids in the membrane, that is, the surface area of the GUV, remains constant during this process (Figure S1). There is no lipid addition. As described in previous theoretical work,^[20]^ the GUV deforms to minimize the energy associated with the line tension at the phase boundary until a bud is connected to the first‐generation vesicle by a tight neck. A common assumption is that the energy barrier for neck scission (i.e. the final pinching of the second‐generation vesicle) is too large to enable vesicle fission without coat proteins. However, while pinching of the lipid constriction comes with an energy cost for opening up the bilayer structure, it also removes the phase boundary.[^21^, ^36^] Therefore, we postulate that complete division could be favorable if the line tension is high enough (Step 3). To implement the proposed division mechanism experimentally, we first need a controlled mechanism to increase the outer osmolarity of the solution. Metabolic processes, that is, the decomposition of molecules through enzymes, inevitably lead to an osmolarity increase. We thus set out to metabolize the sugar solution in which GUVs are often immersed. For this purpose, we make use of the enzyme invertase. Extracellular invertase is secreted by yeast as a form of cell–cell cooperation to decompose sucrose into fructose and glucose (Figure 1 c).^[37]^ We performed osmometer measurements to test if extracellular invertase in a solution of phase‐separated GUVs can produce an increase of the osmolarity ratio *C*/*C*_0_ as required for division. Indeed we find that the osmolarity of the initially 300 mm sucrose solution increases significantly over time (see Figure 1 d). The rate of increase depends on the enzyme concentration. In the presence of 44 mg L^−1^ invertase, the initial osmolarity almost doubles over the course of 150 minutes.

Note that we did not optimize the conditions for invertase activity but chose conditions compatible with the proposed mechanism for GUV division. Phase‐separated GUVs with two distinct hemispheres (Figure 1 e), were successfully electroformed using a lipid mixture consisting of DOPC, cholesterol, DPPC, CL and LissRhod PE (DOPC (18:1 1,2‐dioleoyl‐sn‐glycero‐3‐phosphocholine), DPPC (16:0 1,2‐dipalmitoyl‐sn‐glycero‐3‐phosphocholine), CL (Cardiolipin (Heart, Bovine)), LissRhod PE (18:1 1,2‐dipalmitoyl‐sn‐glycero‐3‐phosphoethanolamine‐N‐(lissamine rhodamine B sulfonyl)); Tables S1 and S2, Mix 1).^[38]^ LissRhod PE labels the ld phase (orange). To test the proposed mechanism for division, we add 44 mg L^−1^ invertase to the GUV‐containing sucrose solution. Figure 1 f shows a time series taken over the course of 45 minutes (see Figure S2 for an overview image with multiple dividing vesicles). We observe the formation of a constriction at the interface of the two phases, eventually leading to complete division. As visible in the final timestep, the second‐generation vesicles diffuse apart as soon as the division is completed, proving that complete neck scission occurred. Control experiments confirm that neither phase separation, nor osmosis alone are sufficient to promote GUV division (Figure S3). To appreciate the continuous deformation process leading to division, Video S1 is recommended. To probe the versatility, we tested twelve additional lipid mixtures and obtain GUVs with two distinct hemispheres from mixtures containing positively, neutral, and negatively charged lipids. Interestingly, we find that the choice of fluorophore attached to the lipid affects the phase separation behaviour (Figure S4, Tables S2–S4). Division was also obtained for GUVs composed from a distinctively different lipid mixture (Table S2, Mix 2, Video S2). We have thus achieved the division of phase‐separated GUVs by increasing the outer osmolarity with an enzymatic reaction. It is interesting to consider that phase separation may have come into play when phospholipids emerged.[^30^, ^31^] By regulating the transcription of a metabolic enzyme like invertase, primitive cells could, in principle, maintain a high level of control over their division without a sophisticated division machinery.

### Theoretical Prediction of the Division Process

**Figure 2:**
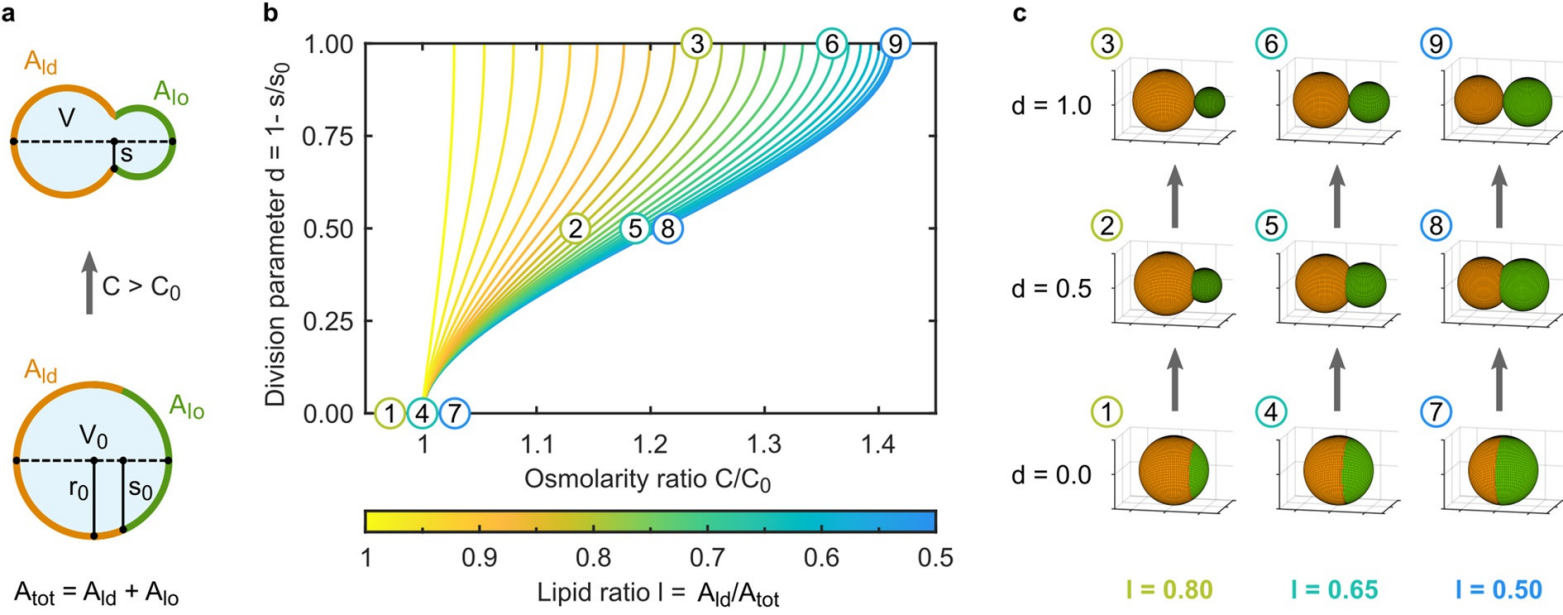
Theoretical predictions for the division process of phase‐separated GUVs based on an increase in the osmolarity ratio. a) Schematic illustration describing the relevant geometrical properties of a deformed GUV (top) and its initially spherical state (bottom). *A_ld_* and *A*_*l*o_ are the surface areas of the spherical caps representing the two phases. *s*_0_ is the radius of the base of the caps, *V*_0_ the volume and *r*_0_ the radius of the initially spherical GUV. *s* is the reduced radius of the base of the caps and V the reduced volume of the deformed GUV. b) Theoretical prediction of the division parameter *d* as a function of the osmolarity ratio *C*/*C*_0_ for different lipid ratios *l. d*=0 corresponds to a spherical GUV, *d*=1 to a fully divided one. c) Predicted shapes of GUVs with different lipid ratios (*l*=0.80, 0.65, 0.50) at defined points during the division process (*d*=0.0, 0.5, 1.0). The corresponding positions (1–9) are indicated in the plot in (b).

To gain control over the process, we set out to predict the osmolarity ratio required to achieve division of a phase‐separated GUV. For this purpose, we develop an analytical model describing the geometrical GUV shape throughout the deformation process as two spherical caps with a base radius *s*_0_ for the initially spherical GUV and *s*<*s*_0_ for the deformed GUV. One of them represents the ld phase with a surface area A_ld_ and the other one the lo phase with a surface area A_lo_, respectively. The relevant geometrical properties (Figure 2 a) can be extracted from confocal images. This representation provides a good approximation of our experimentally observed GUV shapes including a kink at the phase boundary compared to the dumbbell shape expected for single‐phased GUVs. We assume that the total area A_tot_ remains constant throughout the division process. If the outer osmolarity increases (*C*>*C*_0_), the volume of the GUV will decrease due to water efflux. This process is fast compared to the time scale of the division process^[39]^ and therefore assumed to be instantaneous in our model. The equilibrated inner volume is then given by *V*=*C*/*C*_0_ *V*_0_. The resulting excess membrane area allows for deformation of the initially spherical GUV. Deformation minimizes the phase boundary (*s*<*s*_0_) to reduce the energy associated with the line tension.^[20]^ To quantify the progression of the division process, we define a division parameter *d*:

(1) $$d=1-\frac{s}{s_{0}}.$$

*d* is 0 for the initial spherical GUV and 1 for a divided GUV. Based on these geometrical considerations, the osmolarity ratio *C*/*C*_0_ needed to achieve a certain deformation *d* for a symmetric GUV (*A_ld_*=*A*_*l*o_) can be calculated as

(2) $$C/C_{0}=\frac{2}{\sqrt{2-{\left(1-d\right)}^{2}}\left({\left(1-d\right)}^{2}+1\right)}.$$

The model thus postulates that the osmolarity ratio required for complete division (*d*=1) is *C*/*C*_0_=$\sqrt{2}\;$ ≈1.41 (Prediction 1). Since Equation (2) does not depend on the initial radius *r*_0_ of the GUV, the osmolarity ratio required for division is independent of the size of the GUV (Prediction 2). While living cells normally undergo symmetric division, where both second‐generation compartments are of similar size, some processes like oocyte maturation rely on asymmetric division.^[40]^ To extend our model for asymmetric GUVs with $A_{ld}$ ≠$A_{lo}$ we define a lipid ratio parameter $l=A_{ld}/A_{tot}=1-A_{lo}/A_{tot}\;\;$ and hence obtain

(3) $$C/C_{0}=\frac{1}{T_{1}+T_{2}}\mathrm{with}\;\mathrm{the}\;\mathrm{terms}\;T_{1}=\sqrt{l-{\left(1-d\right)}^{2}\left(l-l^{2}\right)}\left(2{\left(1-d\right)}^{2}\left(l-l^{2}\right)+l\right)\mathrm{and}\;T_{2}=\;\sqrt{\left(1-l\right)-{\left(1-d\right)}^{2}\left(l-l^{2}\right)}\;(2{\left(1-d\right)}^{2}(l-l^{2})+(1-l)).$$

See Note S1 in the Supporting Information for a detailed derivation of the equations.

It follows that GUVs with higher asymmetry should require lower osmolarity ratios for complete division and should hence divide faster (Prediction 3). Figure 2 b shows the predicted division parameter d as a function of the osmolarity ratio *C*/*C*_0_ for different lipid ratios *l*. A GUV with *l*=0.8 divides already at an osmolarity ratio of approximately 1.22 (compared to 1.41 for symmetric GUVs with *l*=0.5). For clarity, Figure 2 c displays the predicted shapes of the GUVs corresponding to specific points of the phase space spanned by the division parameter and the osmolarity ratio as indicated in Figure 2 b. Finally, any process that provides a sufficient change in the osmolarity ratio should lead to division of phase‐separated vesicles, independent of the chemical nature of the process (Prediction 4). Compared to previous models describing the shape transformations of lipid vesicles,[^19^, ^20^, ^21^] our model merely considers geometric properties without fitting parameters. Nevertheless, the model yields four predictions, which we will now test experimentally.

### Quantitative Comparison of Experiments and Theoretical Predictions

**Figure 3:**
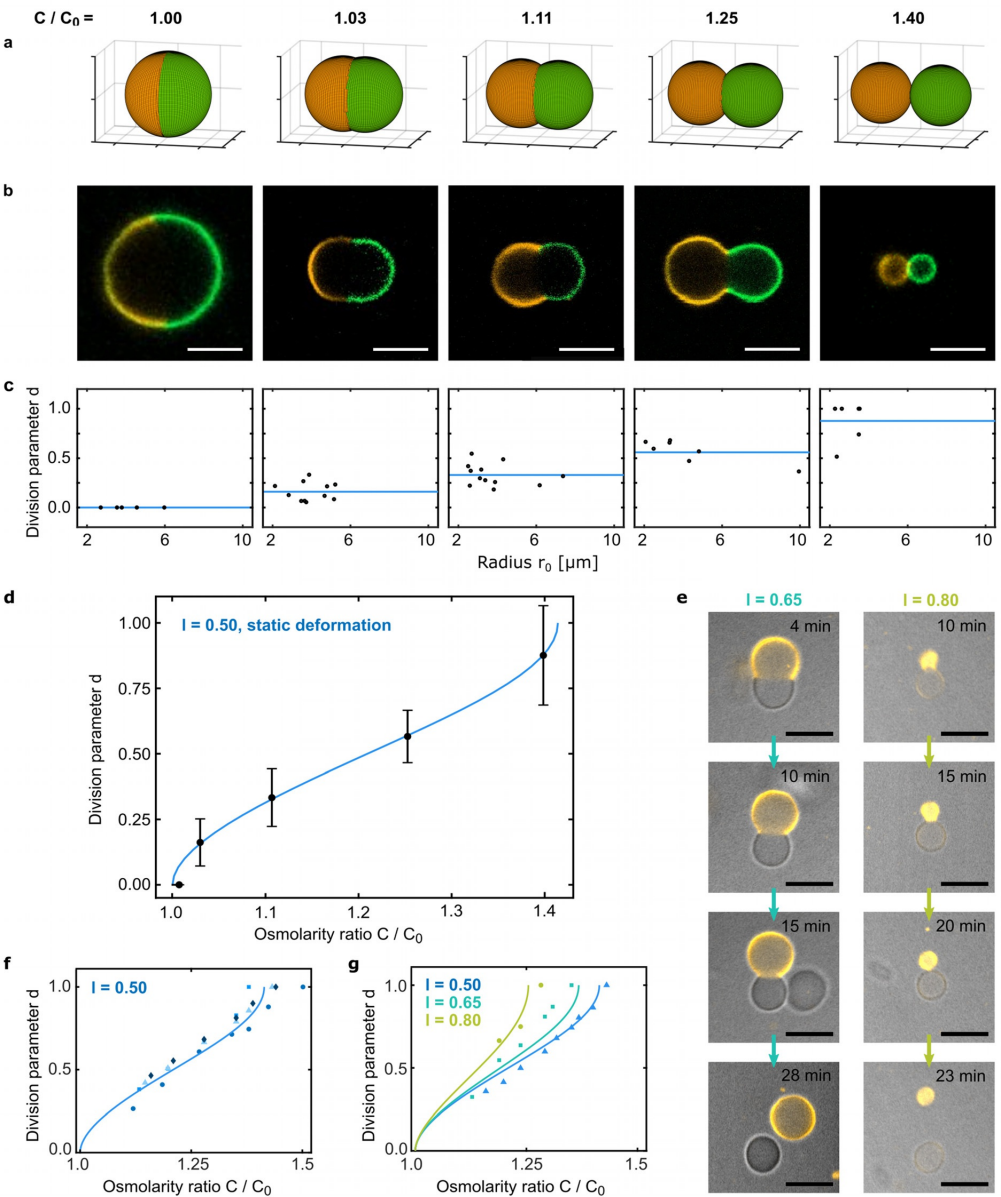
Quantitative comparison of experiment and theoretical prediction. a) Theoretically predicted shapes of symmetric GUVs at different osmolarity ratios *C*/*C*_0_ as indicated. b) Representative confocal fluorescence images of symmetric phase‐separated GUVs immersed in solutions of the corresponding osmolarity ratios *C*/*C*_0_. The ld phase is labeled with LissRhod PE (orange, *λ*_ex_=561 nm), the lo phase with 6‐FAM‐labeled cholesterol‐tagged DNA (green, *λ*_ex_=488 nm). Scale bars: 10 μm. c) Scatter plots of the experimentally determined division parameters plotted against the radius of the initially spherical GUVs. Solid blue lines represent the theoretical prediction, which postulates size‐independence of the division process. d) Division parameter *d* as a function of osmolarity ratio *C*/*C*_0_. The mean values of the measured division parameters (black) and the theoretical prediction from Equation (2) (solid blue line) are plotted. Error bars correspond to the standard deviation of the values for *d* extracted from confocal fluorescence images. e) Confocal fluorescence time series of GUVs with asymmetric lipid ratios (*l*=0.65 and *l*=0.80) in the presence of 44 mg L^−1^ invertase. Scale bars: 10 μm. f) Division parameter *d* of four different symmetric GUVs (*l*=0.5) in the presence of 44 mg L^−1^ invertase plotted against the osmolarity ratio *C*/*C*_0_. The values for *C*/*C*_0_ were obtained from the osmolarity measurements displayed in Figure 1 d. The solid blue line shows the theoretically predicted division curve. g) Division parameter *d* of GUVs with different lipid ratios in the presence of invertase plotted against the osmolarity ratio *C*/*C*_0_. Solid lines are the theoretically predicted division curves for the corresponding lipid ratios.

To test the predictions of our model in a quantitative manner, we first observe symmetric phase‐separated GUVs (*l*=0.5) in solutions with different well‐defined osmolarity ratios *C*/*C*_0_. It is crucial to immerse the GUVs slowly to avoid lipid tubulation (see Figures S2 and S5). To be able to extract geometrical parameters more precisely from the confocal images, we additionally label the lo phase. For this purpose, we add cholesterol‐tagged 6‐FAM‐labeled DNA to the GUVs, which self‐assembles selectively into the lo phase (green) in a Mg^2+^‐containing buffer (see Figure S6). Note that Mg^2+^ leads to a significant reduction of the invertase activity in the presence of GUVs (see Figure S7), likely due to electrostatic interactions between the invertase and the GUVs mediated by divalent ions. Therefore, labelling of the lo phase was omitted for experiments involving invertase. Similarly, we find that in the presence of Mg^2+^ ions, the vesicles remain in close contact after division, again likely due to electrostatic interactions. After soft shaking, they are found in complete isolation (see Figure S8). Figure 3 a shows the theoretically predicted shapes for the different osmolarity ratios. The corresponding representative confocal fluorescence images are presented in Figure 3 b. Note that the shapes are static since the osmolarity ratio is kept constant, unlike in the case of invertase activity. We extract the geometrical parameters required to calculate the division parameter d from multiple images. As postulated, we observe division at an osmolarity ratio of approximately $\sqrt{2}$ (Prediction 1). We find that 90 % percent of the GUVs are single‐phased (*n*=200) at this osmolarity ratio, suggesting a remarkably high division rate. To verify the size independence of the division process (Prediction 2), we used the images of the deformed GUVs to calculate the radius r_0_ of the initially spherical GUV. The scatter plot of the division parameter d over r_0_ is shown in Figure 3 c. As expected, no significant size‐dependent deviations from the theoretical value (blue line) can be observed in the size range of GUVs. For vesicles below 1 μm, size effects and membrane‐specific parameters will likely come into play. As a quantitative comparison of the experimental results with the theoretical prediction [Eq. (2)], we plot the mean division parameter as a function of the osmolarity ratio. Figure 3 d shows that the experimental data agrees well with the theoretical prediction. Deviations may occur due to the fact that GUVs are imaged in solution and can hence rotate in the confocal plane. Trapping can lead to lipid tubulation and hinder the division process (see Figure S9). Note that the quantitative understanding of the vesicle shape as a function of the osmolarity ratio allows us to use phase‐separated GUVs as precise osmolarity sensors. This could for instance be useful for measuring extracellular osmolarity in cell culture based on conventional microscopy without any additional equipment (conventional osmometer measurements require freezing of the sample).

To show that our geometrical description does not only predict the static GUV shapes but also the dynamic division process, we analyse confocal time lapses of the division process in the presence of invertase. We can extract the osmolarity ratio at a given time point from the osmometer measurements in Figure 1 d. Figure 3 f confirms that the division process of symmetric GUVs with two equally large hemispheres (*l*=0.5) agrees well with the prediction. Finally, asymmetric GUVs with *l*>0.5 should require lower osmolarity ratios for division and hence divide faster (Prediction 3). To test this, we observed GUVs with different lipid ratios. Figure 3 e shows that asymmetric GUVs indeed exhibit shorter division times—approximately 27 min for *l*=0.65 and 20 min for *l*=0.80 compared to 40 min for *l*=0.50 (see Figure 1 f). Figure 3 g confirms that the division parameter plotted as a function of the osmolarity ratio follows the theoretical predictions [solid lines, Eq. (3)]. The fact that asymmetric division happens at lower osmolarity ratios may explain why budding was more frequently reported in the literature[^23^, ^24^] than symmetric division.

### Light‐Triggered Local Division

**Figure 4:**
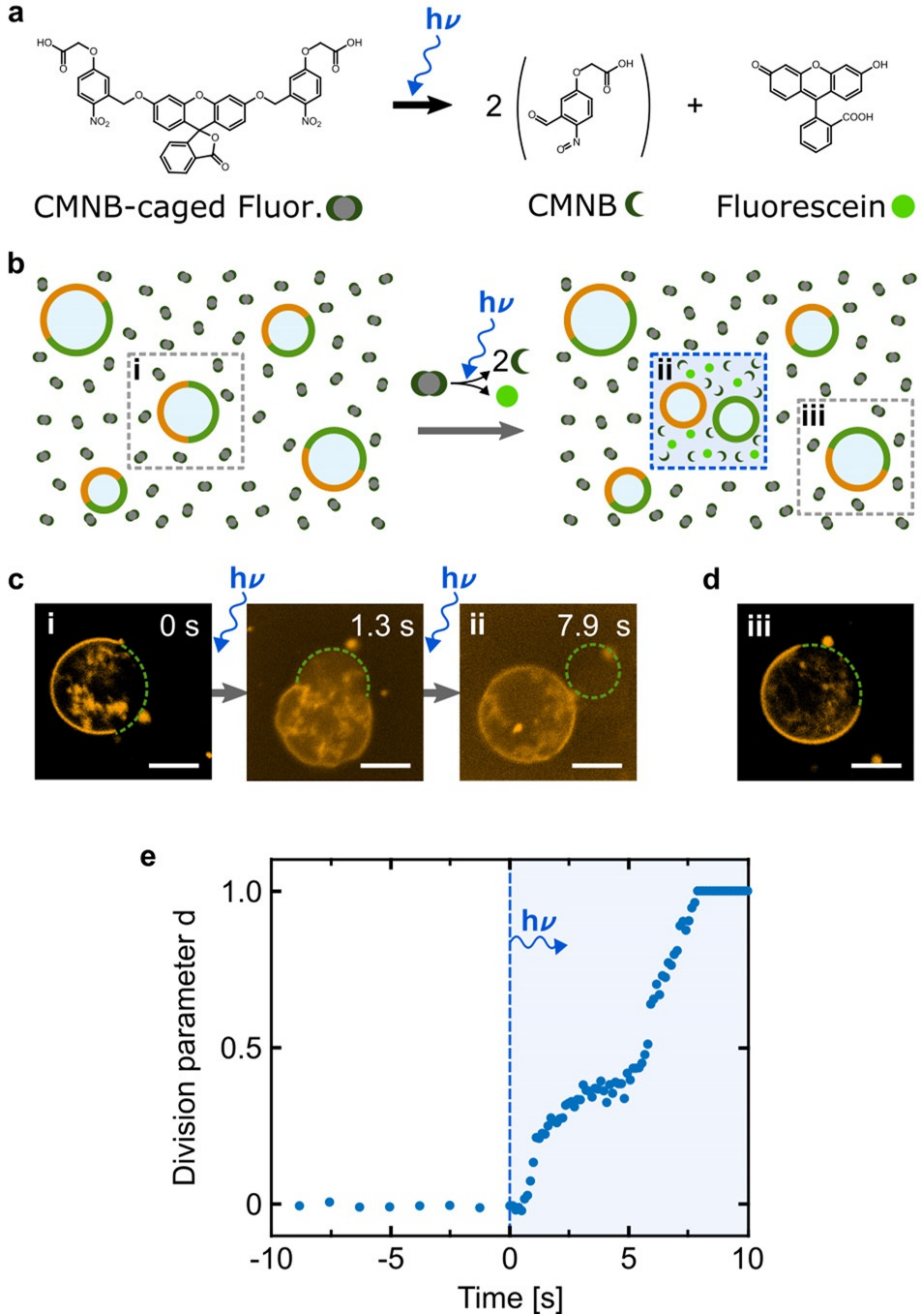
Light‐triggered local division of phase‐separated GUVs via uncaging of CMNB‐fluorescein. a) Chemical reaction pathway of fluorescein release induced by UV or 405 nm illumination. CMNB‐caged fluorescein decomposes into three products thus tripling its contribution to the osmolarity. b) Schematic illustration of the localized light‐triggered division process. Phase‐separated GUVs are immersed in a solution containing CMNB‐fluorescein. Illumination with a 405 nm laser diode leads to a local increase in osmolarity by uncaging of CMNB‐fluorescein and hence to GUV division. c) Representative confocal fluorescence images of a phase‐separated GUV (ld phase labeled with LissRhod PE, *λ*_ex_=561 nm) undergoing full division within seven seconds of 405 nm illumination (time points i and ii are illustrated in b). d) Confocal fluorescence image of a phase‐separated GUV outside the illuminated area maintains its spherical shape (iii as illustrated in b). Scale bars: 10 μm. e) Division parameter d of the GUV shown in (c) over time. The GUV instantly deforms with start of 405 nm illumination (indicated by the vertical blue dashed line) and fully divides within seconds.

Any process that achieves a sufficient increase of the osmolarity ratio should, in principle, be suitable to trigger division of phase‐separated GUVs (Prediction 4). We first demonstrate this by showing that water evaporation can be used instead of invertase activity to increase the osmolarity ratio, see Figure S3 a. This confirms that the division process is not dependent on the chemical nature of the enzymatic reaction but relies on the resulting osmolarity increase. Exploiting this versatility, we want to realize a mechanism with full spatiotemporal control over the division process, such that a selected vesicle divides at a chosen time point whereas surrounding vesicles remain unaffected. We successfully achieve this aim based on the light‐triggered uncaging of bis‐(5‐carboxymethoxy‐2‐nitrobenzyl)‐ether (CMNB)‐caged fluorescein. Upon 405 nm illumination, this initially non‐fluorescent compound splits into three components—two CMNB molecules and the fluorophore fluorescein (Figure 4 a). Its contribution to the overall osmolarity should thus triple. The successful uncaging of fluorescein can be monitored with UV/Vis spectrometry (see Figure S10 a). Figure 4 b illustrates our concept for the localized light‐triggered division: Phase‐separated vesicles are immersed in a solution containing CMNB‐fluorescein. Subsequently, a target GUV is chosen for division. The division process is initiated by illuminating the surrounding area with a 405 nm laser diode leading to uncaging of CMNB‐fluorescein. Fluorescein release increases the osmolarity locally, hence leading to division of the selected GUV, while surrounding GUVs remain unaffected. Based on theoretical considerations (Note S2) and osmometer measurements (Figure S10b), we set the initial concentrations to achieve the required increase of $\sqrt{2}$ in the overall osmolarity. Figure 4 c shows snapshots from a confocal fluorescence time series before (i) and during illumination (ii) of the selected area (Video S3). While division previously happened within tens of minutes (see Figures 1 and 3), the rapid uncaging dynamics of CMNB‐fluorescein promote division after a few seconds. Other representative examples of GUVs undergoing similarly fast division are shown in Figure S11. Note that we could only record one fluorescence track to capture the fast dynamics. The increase in the background fluorescence intensity is due to bleed through from the 405 nm excitation and the release of fluorescein (for confocal images of the fluorescein channel before and after release, see Figure S12). Finally, Figure 4 d highlights the locality of the division: As expected, a vesicle outside the illuminated area does not undergo division. Moreover, illumination alone, in the absence of CMNB‐fluorescein, does not lead to division of phase‐separated GUVs (Figure S13). Figure 4 e plots the division parameter for the vesicle shown in c as a function of time (for more examples see Figure S11). The plot clearly shows that no shape changes occur before illumination (the frame rate was reduced to avoid bleaching and the GUV was observed for in total 100 s before illumination). As soon as the local osmolarity change is induced by uncaging of CMNB‐fluorescein at *t*=0 s, the vesicle starts to deform and fully divides after 7.9 s. The shape of the curve is likely to be a result of the non‐linear increase in osmolarity (Figure S10 b): Uncaging increases the osmolarity locally in the illuminated confocal volume, yet components freely diffuse in and out. Caged compounds have previously been used to change the osmolarity to induce compartment rupture.^[41]^ Here, we have shown that they offer the additional possibility to trigger the division of phase‐separated GUVs locally with light, achieving a rapid time response and division within seconds.

### Regrowth of Phase‐Separated Vesicles After Division

Crucially, synthetic cell division should be followed by a growth phase in order to ultimately sustain multiple growth and division cycles. In our system, this process has to restore the initial phase separation of the GUV. Different methods for vesicle fusion have previously been employed to grow GUVs.[^42^, ^43^, ^44^, ^45^] However, it is not trivial that these conventional fusion mechanisms can lead to phase‐separated GUVs: The emerging line tension adds to the energy barrier for the fusion of a lo‐phase vesicle to a ld‐phase vesicle. As a proof‐of‐principle experiment, we produced carboxyfluorescein‐labeled lo GUVs and rhodamine‐labeled ld GUVs separately, mimicking the single‐phase GUVs after division. With this strategy we can be absolutely sure that a GUV, which contains both fluorescent dyes, results from a fusion event.

We mixed ld and lo GUVs and added Ca^2+^‐ions. This leads to attractive interactions between the GUVs^[46]^ and has been shown to mediate fusion between identical lo‐phase GUVs.^[47]^ We find that this process yields phase‐separated GUVs, which unambiguously demonstrates that fusion between the lo and ld GUVs has occurred (Figure S14, Video S4). It should be noted, however, that despite frequently observed hemifusion and attachment of GUVs to one another, full fusion is a rare event and the vast majority of GUVs (over 95 %) remains single‐phased. Moreover, fusing GUVs again after division cannot lead to growth of the GUV population.

**Figure 5:**
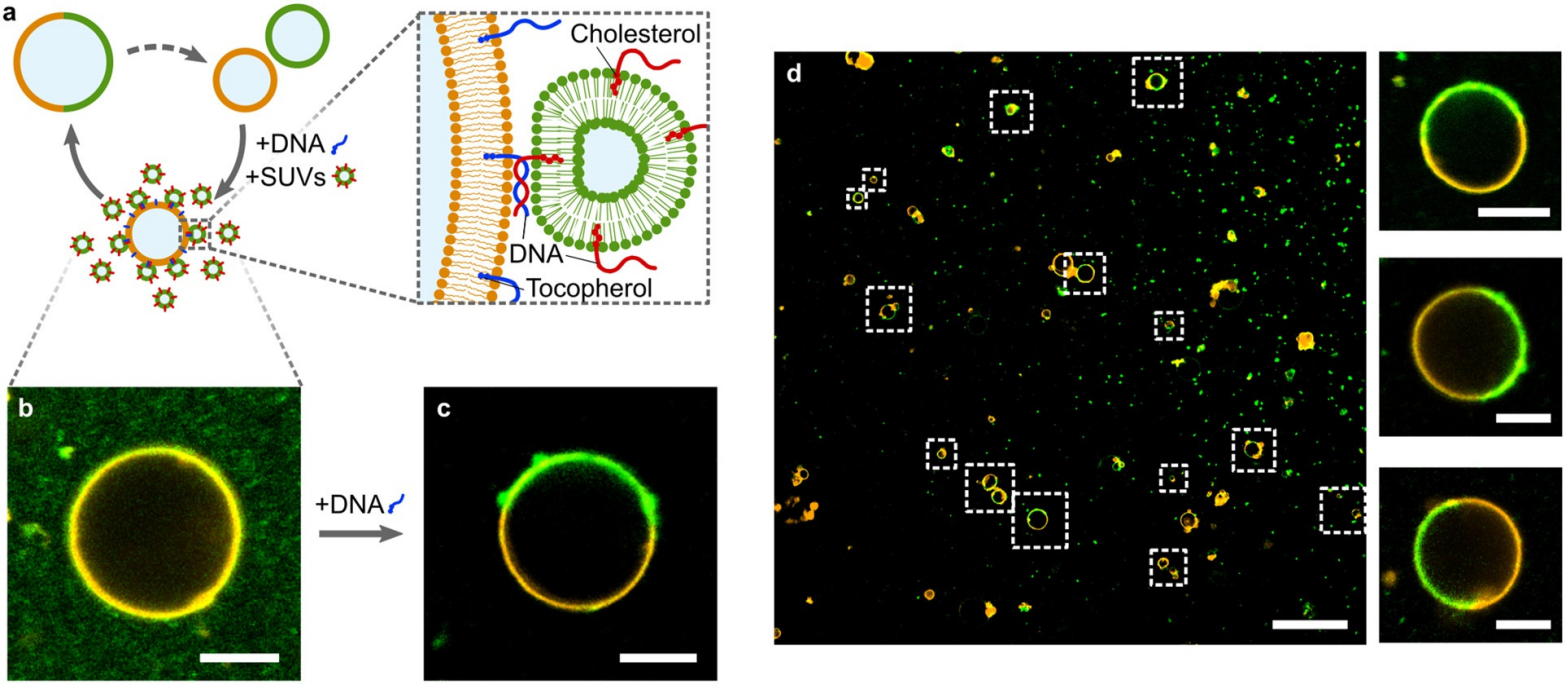
Regrowth of phase‐separated vesicles. a) Schematic illustration of a programmable vesicle growth and division cycle mediated via fusogenic membrane‐bound DNA. The zoom image shows the zipper‐like arrangement of the DNA, bringing the membranes into close proximity. b) Representative confocal fluorescence image of a fluorescently labeled ld‐phase GUV (orange, *λ*_ex_=561 nm) in a feeding bath of lo SUVs functionalized with cholesterol‐tagged 6‐FAM‐labeled DNA (green, *λ*_ex_=488 nm). c) Addition of complementary tocopherol‐tagged DNA leads to SUV fusion and hence the formation of phase‐separated vesicles (as identified via partitioning of cholesterol‐tagged 6‐FAM DNA in presence of unlabeled SUVs). Scale bars: 10 μm. d) Confocal fluorescence overview image (left, scale bar: 50 μm) after the DNA‐mediated fusion process. Fusion took place for the majority of GUVs (highlighted with white boxes). Zoom images (right, scale bars: 10 μm) show the successful regeneration of phase‐separated GUVs with a lipid ratio of *l*≈0.5.

We ultimately need a “feeding mechanism” as illustrated in Figure 5 a, where each growth‐division cycle can increase the total number of GUVs. CaCl_2_‐mediated fusion can restore phase‐separation upon addition of small unilamellar vesicles (SUVs) to GUVs (Figure S15). However, this approach lacks programmability. In order to achieve targeted fusion of SUVs to GUVs of the, respectively other lipid phase in a mixture, we thus make use of the sequence‐programmable base‐pairing of DNA. As we already demonstrated, cholesterol‐tagged DNA self‐assembles selectively into the liquid‐ordered phase. We find that in our system tocopherol‐tagged DNA, on the other hand, attaches to both phases equally (Figure S16). By designing complementary single‐strands of DNA, one with a 3′ cholesterol and the other one with a 5′ tocopherol, we can thus selectively bring vesicle membranes into close proximity as illustrated in the zoom in Figure 5 a. Such zipper‐like DNA‐based mimics of SNARE proteins have been used to trigger fusion of SUVs of the same kind,^[44]^ but it is not trivial that phase‐separated vesicles can be formed. We hence immersed ld GUVs in a feeding bath containing DNA‐functionalized lo SUVs (Figure 5 b). Note that the SUVs (green) with a diameter of around 100 nm (Figure S17) are too small to be resolved individually. Upon addition of the complementary DNA, we observe phase‐separated GUVs with a sufficiently large lo phase to restore the initial condition. Given the area of the ld phase in Figure 5 c, we estimate that approximately 5600 SUVs have fused to the GUV. We hypothesize that the line tension at phase boundary present after the first fusion event lowers the energy barrier for subsequent fusion. Lipid phase boundaries have been shown to promote other fusion events including HIV entry.^[48]^ The time‐resolved growth process is depicted in Figure S18. Note that the SUVs have a larger surface‐to‐volume ratio compared to the GUVs and thus are supplied in a lower osmolarity solution in order to obtain spherical GUVs after fusion. The small osmolarity mismatch is likely to be beneficial for the fusion process itself.^[15]^ By preventing duplex formation, that is, in absence of tocopherol‐tagged DNA, we do not observe vesicle fusion (Figure S19). Compared to Ca^2+^ mediated fusion, we did not only gain programmability, but also increased the efficiency of the process. Figure 5 d shows an overview image, which demonstrates that phase‐separation was restored in the majority of GUVs after incubation. A lipid ratio of *l*≈0.5 could be restored reproducibly.

## Conclusion

Synthetic cell division is one of the most exciting albeit challenging tasks towards the bottom‐up construction of cellular systems. Our study realizes the division of GUVs, fully controllable by two physical parameters—phase separation and osmosis. Phase separation of the lipids in the GUV membrane defines the plane of division such that an increase of the surface‐to‐volume ratio by osmosis leads to contraction at the phase boundary and thus the formation of two second‐generation compartments. We derived a model of the division process based on geometrical considerations. The analytical model makes four predictions, which were all verified experimentally: First of all, the osmolarity ratio required for division of GUVs with equally sized phases is $\sqrt{2}$ ; secondly, the time‐point of division is independent of vesicle size; third, asymmetric division happens faster (i.e. at lower osmolarity ratios) and fourth, any process, which leads to a sufficiently large osmolarity increase, can trigger division. We showcased the latter by demonstrating division as a result of fundamentally distinct processes, including water evaporation, metabolic decomposition of sugars and light‐triggered uncaging of CNMB‐fluorescein. Using light as a stimulus for division provides full spatiotemporal control, which could, in the future be exploited to perform directed evolution of a vesicle population. The concept to exploit caged compounds for local vesicle division is new and broadly applicable. It does not rely on specific environmental conditions and can directly be extended from CMNB‐caged fluorescein to other caged compounds. Any suitable division mechanism for synthetic cells should have the capacity to sustain multiple growth‐and‐division cycles. In our case, growth has to restore phase separation in the second‐generation compartments. We achieve fusion of SUVs of the other phase to single‐phased GUVs with programmable DNA‐based SNARE protein mimics—thus restoring the initial conditions for subsequent division cycles, which will undoubtedly be a prerequisite for the evolution of synthetic cellular systems. The future integration of information storage and replication will be yet another important milestone towards the visionary transition from matter to life, or, in other words, towards a synthetic cell which truly deserves its name. In the meantime, our engineering approach to synthetic cell division prompts questions about cellular life as we know it: We may be curious to discover whether phase separation and osmosis may have sustained compartment division at the onset of life, possibly regulated by the expression of metabolic enzymes. And we may further ask how remnants thereof play a role in cell biology today—continuously nurturing the emergence of cells from cells.

## Conflict of interest

The authors declare no conflict of interest.

## Supporting information

As a service to our authors and readers, this journal provides supporting information supplied by the authors. Such materials are peer reviewed and may be re‐organized for online delivery, but are not copy‐edited or typeset. Technical support issues arising from supporting information (other than missing files) should be addressed to the authors.

Supplementary

Supplementary

Supplementary

Supplementary

Supplementary
