# Supplementary material for: Division and Regrowth of Phase‐Separated Giant Unilamellar Vesicles
Source: Angew Chem Int Ed Engl. 2021 Mar 24;60(19):10661–9. doi: 10.1002/anie.202014174 (PMC8252472; doi:10.1002/anie.202014174)
Supplement: Supplementary file 1 — Supplementary [file ANIE-60-10661-s002.pdf]

Angewandte Chemie
Eine Zeitschrift der Gesellschaft Deutscher Chemiker

Supporting Information

Division and Regrowth of Phase-Separated Giant Unilamellar Vesicles**

*Yannik Dreher+, Kevin Jahnke+, Elizaveta Bobkova, Joachim P. Spatz, and Kerstin Göpfrich**

anie_202014174_sm_miscellaneous_information.pdf
anie_202014174_sm_VideoS1.mp4
anie_202014174_sm_VideoS2.mp4
anie_202014174_sm_VideoS3.mp4
anie_202014174_sm_VideoS4.mp4

Contents

1 Materials and Methods **4**

1.1 GUV formation . . . 4
1.2 SUV formation . . . 5
1.3 Confocal fluorescence microscopy . . . 5
1.4 Theoretical predictions . . . 5
1.5 Determination of osmolality . . . 6
1.6 Enzymatic osmolarity change . . . 6
1.7 Light-mediated osmolarity change . . . 6
1.8 Absorbance measurements . . . 7
1.9 Calcium-mediated vesicle fusion . . . 7
1.10 DNA-mediated vesicle fusion . . . 7

2 Supporting Tables **8**

2.1 Table S1: List of used lipids . . . 8
2.2 Phase separation of GUVs produced from different lipid mixtures . . . 9
2.2.1 Table S2: Influence of lipid type on phase separation . . . 9
2.2.2 Table S3: Influence of lipid charge on phase separation . . . 10
2.2.3 Table S4: Influence of the fluorescently-labeled lipid on phase separation . . . 11
2.3 Table S5: Electroformation protocol for phase-separated GUVs . . . 12
2.4 Table S6: Electroformation protocol for single-phased GUVs . . . 12

3 Supporting Figures **13**

3.1 Figure S1: Area, volume and surface-to-volume ratio over time . . . 13
3.2 Figure S2: Overview confocal image and lipid tubulation . . . 14
3.3 Figure S3: GUV division upon water evaporation . . . 15
3.4 Figure S4: Phase separation of GUVs produced from different lipid mixtures 16
3.5 Figure S5: Osmolarity mismatch after electroformation . . . 17
3.6 Figure S6: Necessity of $MgCl_2$ for attachment of cholesterol-tagged DNA . 18
3.7 Figure S7: Reduction of invertase activity in the presence of $MgCl_2$ . . . 19
3.8 Figure S8: Gentle shaking overcomes $MgCl_2$-mediated electrostatic interaction between divided GUVs . . . 20
3.9 Figure S9: Microfluidic trapping approach . . . 21
3.10 Figure S10: Characterisation of CMNB-caged fluorescein via absorbance and osmolarity measurements . . . 23
3.11 Figure S11: Light-triggered division of phase-separated GUVs via uncaging of CMNB-caged fluorescein . . . 24
3.12 Figure S12: Illumination of CMNB-fluorescein with a 405 nm laser diode leads to uncaging of fluorescein . . . 26

3.13 Figure S13: 405 nm illumination in absence of CMNB-caged fluorescein does not lead to division of phase-separated GUVs 27
3.14 Figure S14: Phase separation can be restored through Ca^{2+}-mediated fusion of single-phased GUVs 28
3.15 Figure S15: Phase separation can be restored through Ca^{2+}-mediated fusion of SUVs to single-phased GUVs 29
3.16 Figure S16: Tocopherol-tagged DNA is homogeneously distributed in the lo- and ld-phases of phase-separated GUVs 30
3.17 Figure S17: DLS of lo SUVs 31
3.18 Figure S18: DNA-mediated vesicle fusion leads to growth of lo-phase of initially single-phased GUVs 32
3.19 Figure S19: DNA-functionalized lo SUVs do not fuse to plain ld GUV . . . 33

4 Supporting Notes **34**
4.1 Note S1: Derivation of theoretical prediction 34
4.2 Note S2: Considerations for osmolarity matching with CMNB-caged fluorescein 35
4.3 Note S3: Osmolarity vs. osmolality 36

5 Supporting Videos **37**
5.1 Video S1: Conceptual model and confocal fluorescence time lapse of GUV division 37
5.2 Video S2: Division of phase-separated GUV triggered by enzymatic decomposition 37
5.3 Video S3: Light-triggered division of phase-separated GUVs by uncaging of CMNB-fluorescein 38
5.4 Supporting Video S4: Calcium-mediated fusion of single-phase GUVs to restore phase-separation 38

Supporting References **39**

1 Materials and Methods

1.1 GUV formation

18:1 Atto488-DOPE was purchased from ATTO-TEC GmbH. All other lipids were purchased from Avanti Polar Lipids, Inc., and stored in chloroform at −20 °C (for a complete list of used lipids see Table S1). Giant unilamellar vesicles (GUVs) were produced via the electroformation method [1] using a VesiclePrepPro device (Nanion Technologies GmbH). Four types of GUVs were produced for the experiments shown in main. Phase-separated GUVs (Table S2, Mix 1) are composed of 27.125 % 18:1 DOPC, 24,75 % cholesterol, 37.125 % 16:0 DPPC, 10 % cardiolipin (CL), 1 % 18:1 LissRhod PE if not stated otherwise. Single-phase GUVs (Mix SP1) are composed of 70 % EggPC, 29 % EggPG, 1 % 18:1 Atto488-DOPE, whereas lo single-phase GUVs are composed of 49,5 % 18:1 DOPC, 49,5 % cholesterol, 1 % CF PE (lo Mix) and ld single-phase GUVs are composed of 4,75 % 18:1 DOPC, 74.25 % 16:0 DPPC , 20 % cardiolipin (CL), 1 % 18:1 LissRhod PE (ld Mix). Note that DPPC membranes containing 20 mol% CL were shown to exhibit a ld phase [2]. Furthermore, addition of $<$ 10 mol% of DOPC disrupts the order of DPPC membranes [3]. To test the phase separation behavior of GUVs, vesicles from twelve different additional lipid mixtures were prepared, for details see Tables S2-S4. 40 µl of 1 mM lipid mix in $CHCL_3$ were homogeneously spread on the conductive side of an indium tin oxide (ITO) coated glass coverslide (Visiontek Systems Ltd) using a cover slide. The lipid-coated ITO slide was subsequently placed under vacuum for at least 30 min to achieve complete evaporation of the $CHCL_3$. A rubber ring with a diameter of 18 mm was placed on the lipid-coated ITO slide. The ring was filled with 275 µl buffer solution, before creating a sealed chamber by placing a second ITO slide on top. The buffer solution used for the phase-separated GUVs contained 300 mM sucrose (Sigma-Aldrich Corp.) and 10 mM HEPES (Sigma-Aldrich Corp.). The solution for the lo and ld single-phase GUVs (lo Mix and ld Mix) contained 300 mM sucrose. Both solutions were preheated to 65 °C. For the single-phase GUVs (Mix SP1) a 500 mM sucrose solution at room temperature was used. The assembled electroformation chamber was placed into the VesiclePrepPro and connected to the electrodes. A programmable AC field was applied across the ITO slides. For the phase-separated GUVs, a custom-written multi-step program with defined temperature, voltage, AC-frequency and duration was used (see Table S5). For both single-phase GUVs the preinstalled *Standard* program was selected (see Table S6). GUVs were collected immediately after formation according to the protocol and stored at 4 °C for up to 2 days.

1.2 SUV formation

Small unilamellar vesicles (SUVs) composed of 49,5 % 18:1 DOPC, 49,5 % cholesterol, 1 % CF PE (lo Mix) were formed by mixing the lipids dissolved in $CHCl_3$in a glass vial and subsequent solvent evaporation under a stream of nitrogen gas. The glass vial was then placed under vacuum for at least 30 min to remove residual traces of solvent. A solution of 300 mM sucrose was preheated to 65 °C and added to the glass vial to resuspend the lipids at a concentration of 3 mM. After 10 min swelling at 65 °C the solution was vortexed at 1000 rpm for at least 5 min to trigger liposome formation. Homogenous SUVs were formed by extruding the liposome solution nine times through a polycarbonate filter with a pore size of 100 nm (Avanti Polar Lipids, Inc.). During this step the extruder was heated to 65 °C using a heating plate. The SUVs were stored at 4 °C for up to 5 days.

1.3 Confocal fluorescence microscopy

A confocal laser scanning microscope LSM 800 or LSM 880 (Carl Zeiss AG) was used for fluorescence imaging. The images were acquired using a 20x air (Objective Plan-Apochromat 20x/0.8 M27, Carl Zeiss AG) and a 40x water immersion objective (LD C-Apochromat 40x/1.1 W, Carl Zeiss AG). To visualize the phase separation of the GUVs, 6-FAM-labelled cholesterol-tagged DNA (Integrated DNA Technologies, Inc.; DNA sequence: 5' 6-Fam-CTATGTATTTTGCACAGTTT-Chol 3'; HPLC purified, DNA #1) was used which partitioned mainly into the lo phase [4]. LissRhod PE labelled the ld phase. 6-FAM and Atto488-DOPE were excited with a 488 nm diode laser (Carl Zeiss AG), LissRhod PE with a diode or Argon laser at 561 nm (Carl Zeiss AG). In order to release the fluorescein for the light-triggered division, CMNB-caged fluorescein was illuminated with a diode laser at 405 nm. Images were analyzed with ImageJ (contrast and brightness adjustments and TrackMate) and Matlab.

1.4 Theoretical predictions

All calculations were carried out with MathWorks Matlab (9.5.0.944444 R2018b) and Jupyter (v. 4.4.0) as described in the main text. The derivation of the equations is shown in Supplementary Note S1.

1.5 Determination of osmolality

The osmolality of all solutions was measured with the Osmomat 030 (Gonotec GmbH). Before use, the osmometer was calibrated with calibration solutions of 0 , 300 and 900 mOsm/kg (Gonotec GmbH). Each measurement was carried out with a sample volume of 50 µl. Note that for the quantities that are calculated here, the osmolality is a good approximation for the osmolarity (see Note S3). The measurement error of the osmometer itself (in terms of reproducibility) is below 0.5%.

1.6 Enzymatic osmolarity change

Invertase from bakers's yeast (S. cerevisiae) grade VII, $\geq$ 300 units/mg was purchased from Sigma-Aldrich Corp. Nominally, one unit of the enzyme hydyrolyzes 1 µmol of sucrose per minute to produce fructose and glucose at pH 4.5 at 55 °C. The GUV solution obtained from electroformation was mixed with a solution containing 1 mg ml^{-1} invertase such that the final concentration of invertase was 44.4 mg l^{-1} and in a second experiment 22.2 mg l^{-1}. Immediately after mixing, a part of the solution was used for division experiments with phase-separated GUVs in a sealed observation chamber. The other part was used for osmolality measurements over time. The experiments were carried out at room temperature.

1.7 Light-mediated osmolarity change

CMNB-caged fluorescein (Fluorescein bis-(5-Carboxymethoxy-2-Nitrobenzyl)-Ether, dipotassiumsalt) was purchased from Sigma-Aldrich Corp., dissolved in 100 mM Tris buffer at pH 8.0 at a final concentration of 50 mM and stored at −20 °C until use. Phase-separated GUVs were produced in 13 mM sucrose and mixed with an osmolarity-matched solution of CMNB-caged fluorescein leading to a final concentration of 1.73 mM CMNB-caged fluorescein. Note that the concentration of the sucrose solution was reduced to ensure a sufficiently large osmolarity change despite the limited concentration of the caged fluorescein (see Note S2). Each phase-separated GUV was imaged with confocal fluorescence microscopy for at least 100 s before illumination with 405 nm laser light (70 % laser intensity) leading to the uncaging of CMNB-caged fluorescein and hence a local increase in osmolarity.

1.8 Absorbance measurements

The absorbance of CMNB-caged fluorescein was measured with a plate reader (TECAN microplate reader SPARK®) in the range from 300-700 nm using a 96-well plate. The settle time was set to 50 ms. In order to induce uncaging of CMNB-fluorescein, the solution was transferred into a PCR-tube, diluted with MQ and put under a UV-lamp (Hamamatsu LIGHTNINGCURE Spot light source LC8, 100 % power). Subsequently, the solution was pipetted into the well-plate again and measured for a second time.

1.9 Calcium-mediated vesicle fusion

Ld-phase GUVs (20 % CL, 74.25 % DPPC, 4.75 % DOPC, 1 % LissRhod PE) and lo-phase SUVs or GUVs (49.5 % DOPC, 49.5 % cholesterol, 1 % CF PE) at lipid concentrations of ~ 0.3 mM were mixed in a 1:1 ratio. Note that the lipid mix used for lo GUVs was shown to exhibit a homogeneous lo phase [5, 6]. The ld composition [3, 2] was chosen in order to obtain the initial phase-separated lipid mixture after fusion. A second type of GUVs, which we refer to as ld GUVs [3, 2], was formed, such that we obtain the initial phase-separated lipid mixture after fusion. After addition of isoosmolar $CaCl_2$ at a final concentration of 20 mM, the vesicle-containing solution was observed with a confocal fluorescence microscope in a sealed observation chamber. After > 10 min the first phase-separated GUVs containing both fluorescent dyes could be observed.

1.10 DNA-mediated vesicle fusion

In order to obtain DNA-mediated vesicle fusion we designed two complementary DNA sequences, adapted from [7], that arrange in a zipper like manner upon duplex formation, thereby bringing the membranes of two distinct vesicles in close proximity. Lo-phase SUVs (50 % DOPC, 50 % cholesterol) were incubated with 1 µM cholesterol-tagged DNA (Biomers; DNA sequence: 5' TGGACATCAGAAAGGCACGACGA-Chol 3'; HPLC purified, DNA #2) and 10 mM $MgCl_2$ for 20 min to minimize the amount of unbound DNA. On the other hand, ld-GUVs (20 % CL, 74.25 % DPPC, 4.75 % DOPC, 1 % LissRhod PE) were incubated with 1 µM tocophorol-tagged DNA (from Biomers; DNA sequence: 5'-Toc TCCGTCGTGCCTTATTTCTGATGTCCA 3'; HPLC purified, DNA #3), which inserts into lo- and ld-lipid phases (see Figure S15). After incubation, lo-SUVs and ld-GUVs were mixed and observed with a confocal fluorescence microscope. To prove the successful regrowth of phase-separated GUVs, cholesterol-tagged 6-FAM DNA (Integrated DNA Technologies, Inc.; DNA sequence: 5' 6-Fam-CTATGTATTTTGCACAGTTT-Chol 3'; HPLC purified, DNA #1) was added to visualize the lo-phase as described previously.

2 Supporting Tables

2.1 Table S1: List of used lipids

Lipid	Abbreviation	Company
18:1 1,2-dioleoyl-sn-glycero-3-phosphocholine	DOPC	Avanti Polar Lipids, inc.
16:0 1,2-dipalmitoyl-sn-glycero-3-phosphocholine	DPPC	Avanti Polar Lipids, inc.
18:1 1,2-dioleoyl-3-trimethylammonium-propane	DOTAP	Avanti Polar Lipids, inc.
18:1 1,2-dioleoyl-sn-glycero-3-phospho-(1'-rac-glycerol)	DOPG	Avanti Polar Lipids, inc.
18:1 1,2-dioleoyl-sn-glycero-3-phosphoethanolamine	DOPE	Avanti Polar Lipids, inc.
cholesterol (ovine)	Chol	Avanti Polar Lipids, inc.
18:0 N-stearoyl-D-erythro-sphingosylphosphorylcholine	SM	Avanti Polar Lipids, inc.
Cardiolipin (Heart, Bovine)	CL	Avanti Polar Lipids, inc.
L-α-phosphatidylcholine	EggPC	Avanti Polar Lipids, inc.
L-α-phosphatidylglycerol	EggPG	Avanti Polar Lipids, inc.
18:1 1,2-dioleoyl-sn-glycero-3-phosphoethanolamine-N-(Cyanine 5)	Cy5 PE	Avanti Polar Lipids, inc.
18:1 1,2-dipalmitoyl-sn-glycero-3-phosphoethanolamine-N-(lissamine rhodamine B sulfonyl)	LissRhod PE	Avanti Polar Lipids, inc.
18:1 1,2-dioleoyl-sn-glycero-3-phosphoethanolamine-N-(carboxyfluorescein)	CF PE	Avanti Polar Lipids, inc.
16:1 1-palmitoyl-2-6-[(7-nitro-2-1,3-benzoxadiazol-4-yl)amino]hexanoyl-sn-glycero-3-phosphocholine	NBD PC	Avanti Polar Lipids, inc.
18:1 1,2-dioleoyl-sn-glycero-3-phosphoethanolamine-N-(7-nitro-2-1,3-benzoxadiazol-4-yl)	NBD PE	Avanti Polar Lipids, inc.
18:1 Atto488-labelled 1,2-Dioleoyl-sn-glycero-3-phosphoethanolamin	Atto488-DOPE	ATTO-TEC GmbH

Supporting Table S1
List of all lipids used in this study and their abbreviations.

2.2 Phase separation of GUVs produced from different lipid mixtures

2.2.1 Table S2: Influence of lipid type on phase separation

Mix No.	Lipid mixture	Phase separation
1	27.1% DOPC, 24.8% chol, 37.1% DPPC, 10% CL + 1% LissRhod PE	yes
2	37.1% DOPC, 24.8% chol, 37.1% DPPC + 1% LissRhod PE	yes
3	33 % DOPC + 33 % SM + 33 % chol + 1 % NBD PC	yes

Supporting Table S2
Overview of the influence of lipid type on phase separation behaviour of GUVs. All GUVs were prepared using a 300 mM sucrose solution and the electroformation method with the protocol presented in Supporting Table S5. For all mixes we obtained phase-separated GUVs with distinct hemispheres. For confocal fluorescence images of the GUVs see Figure S2.

2.2.2 Table S3: Influence of lipid charge on phase separation

Mix No.	Lipid type [mol % / lipid charge]	Additions	Phase separation
2	DOPC [37.1 %/no]	-	yes
1	DOPC [27.1 %/no]<br>CL [10 %/-]	-	yes
4	DOTAP [27.1 %/+]<br>CL [10 %/-]	-	yes
5	DOTAP [27.1 %/+]<br>CL [10 %/-]	unlabeled DOPE instead of LissRhod PE,<br>Chol-Atto390-DNA<br>10mM $MgCl_2$	yes
6	DOPG [27.1 %/-]<br>CL [10 %/-]	-	yes

Supporting Table S3
Overview of the influence of lipid-charge on phase separation of GUVs. All presented lipid mixtures contain 24.8 % cholesterol, 37.1 % DPPC and 1 % LissRhod PE, supplemented with other lipids as listed in the table. The presented charges apply for physiological conditions. All GUVs were prepared using a 300 mM sucrose solution and the electroformation method with the protocol presented in Supporting Table S5. Electroformation of Lipid Mix 6 did not yield GUVs and GUVs from this mix were, therefore, prepared via gentle hydration for two hours at 65 °C [8]. Phase-separated GUVs with two distinct hemispheres were obtained using neutral as well as positively or negatively charged lipids. For confocal fluorescence images of the GUVs see Figure S2.

2.2.3 Table S4: Influence of the fluorescently-labeled lipid on phase separation

Mix No.	Fluorophore	Additions	Phase separation
1	1 % LissRhod PE	-	yes
7	1 % LissRhod PE	Chol-6-FAM-DNA + 10mM $MgCl_2$	yes
8	1 % Cy5-PE	-	yes
9	0.5 % Atto488-DOPE + 0.5 % LissRhod PE	-	yes
10	1 % NBD PE	-	no
11	0.5 % NBD PE + 0.5 % Cy5 PE	-	yes
12	1 % DOPE (no fluorophore)	Chol-6-FAM-DNA + 10mM $MgCl_2$	no
13	0.5 % LissRhod PE + 1 % CF PE	-	yes

Supporting Table S4
Overview of the influence of the fluorophore on phase separation of GUVs. All listed lipid mixtures contained 27.1 % DOPC, 24.8 % cholesterol, 37.1 % DPPC, 10 % cardiolipin and 1 % of the labeled lipid as specified in the table. In all cases, the fluorophore was covalently linked to the lipid head group. All GUVs were prepared using a 300 mM sucrose solution and the electroformation method with the protocol presented in Supporting Table S5. It is important to note that the choice of fluorphore alone can influence the phase-separation behaviour of the GUVs, see Mix 10 and 12 (no phase-separation). For confocal fluorescence images of the GUVs see Figure S2.

2.3 Table S5: Electroformation protocol for phase-separated GUVs

**Step**	**Time [s]**	**Ampl [V]**	**Freq [Hz]**	**Temp [°C]**
Initiate	300	1	10	70
Main	2100	1	10	70
Detach1	2160	1	3	70
Detach2	3000	1	3	70
Detach3	3060	1	1	70
Detach4	3420	1	1	70
Detach5	3480	1	0.5	70
Detach6	3840	1	0.5	70
Detach7	3960	1	0	70

Supporting Table S5
Electroformation protocol for the formation of phase-separated GUVs using the Vesicle Prep Pro (Nanion Technologies GmbH). Custom-written multi-step program for formation of phase-separated GUVs, adapted from a previously published protocol [4]. Note that it is crucial to keep the temperature above the phase-transition temperature throughout the electroformation. Parameters are changed linearly over time from one step to the next.

2.4 Table S6: Electroformation protocol for single-phased GUVs

**Step**	**Time [s]**	**Ampl [V]**	**Freq [Hz]**	**Temp [°C]**
Initiate	180	3	5	37
Main	7380	3	5	37
Detach	7680	0	5	37

Supporting Table S6
Electroformation protocol for single-phase GUV formation using the Vesicle Prep Pro (Nanion Technologies GmbH). The programme was preinstalled as the standard protocol for GUV formation. Parameters are changed linearly over time from one step to the next.

3 Supporting Figures

3.1 Figure S1: Area, volume and surface-to-volume ratio over time

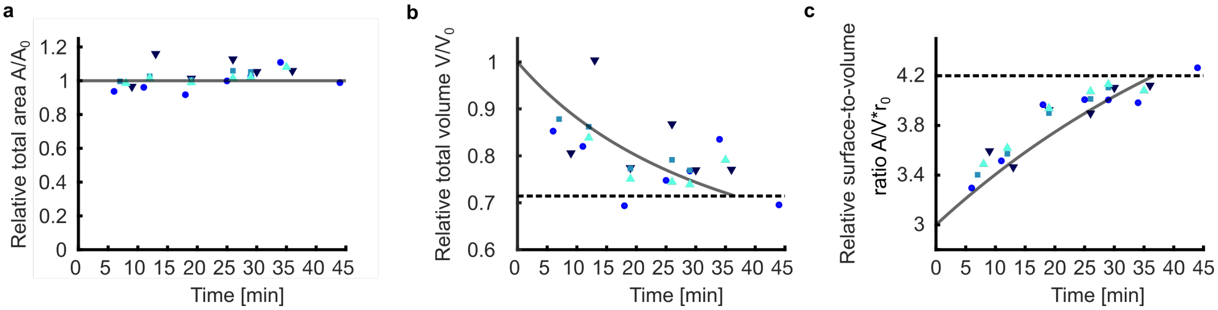

Supporting Figure S1

Relative area, volume and surface-to-volume ratio of phase-separated vesicles over time. The data was extracted from the same time series as the plot in Figure 3**f** of the main text. **a, b, c** triangles, circles and squares represent different vesicles with $l = 0.5$ in the presence of invertase, solid grey line shows the expected curve as calculated from osmolarity measurements. Dashed black line shows the critical volume ratio **b** or surface-to-volume ratio **c** at which full division should occur. As assumed in our model, the surface area remains constant over time, while the surface-to-volume ratio increases.

3.2 Figure S2: Overview confocal image and lipid tubulation

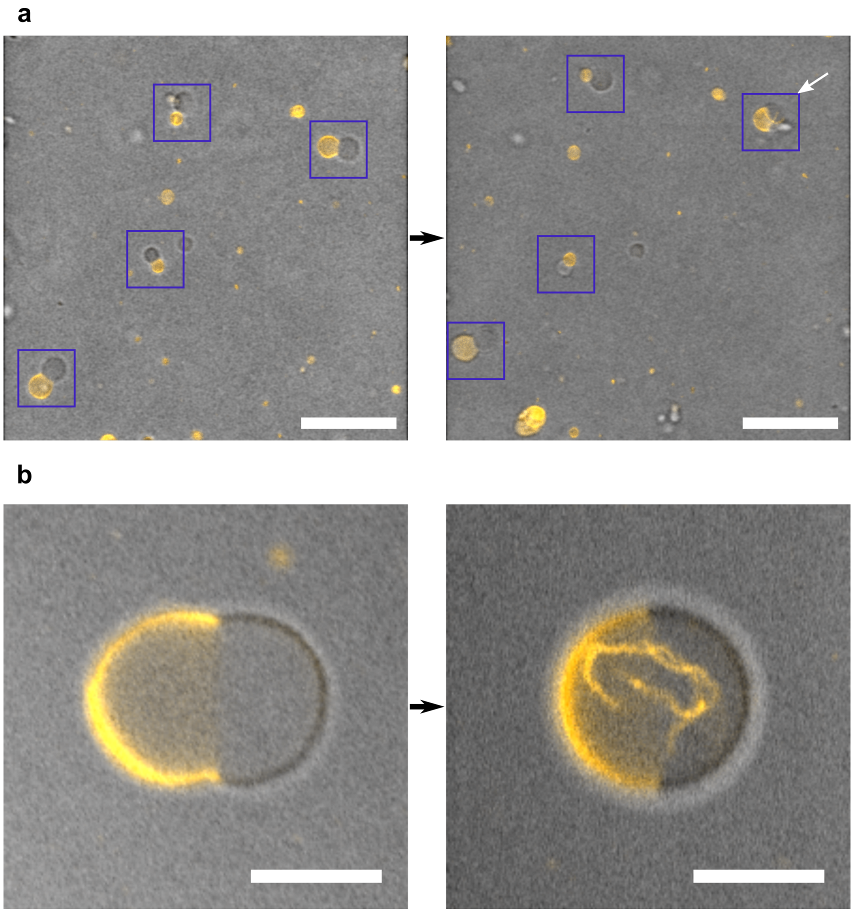

Supporting Figure S2
Reproducibility of the division process. **a** Overlays of confocal (ld phase labeled with LissRhod PE, $\lambda_{ex} = 561\,\mathrm{nm}$) and bright field images of multiple vesicles undergoing division due to a continuous increase of the outer osmolarity due to invertase activity. The white arrow highlights a vesicle that restores it spherical shape after the formation of lipid tubes – potentially due to interactions with the glass surface or too fast local osmolarity change. Scale bars: 25 µm **b** Zoom image of a vesicle which shows tubulation. The GUV deforms initially (left image). As the osmotic pressure increases over time, lipid tubulation is observed and the spherical shape is restored (right image). This effect inhibits successful division of the GUVs. This occurred mainly when the osmolarity was too high, if it changed too quickly or if the GUV was in contact with surfaces. Scale bars: 10 µm.

3.3 Figure S3: GUV division upon water evaporation

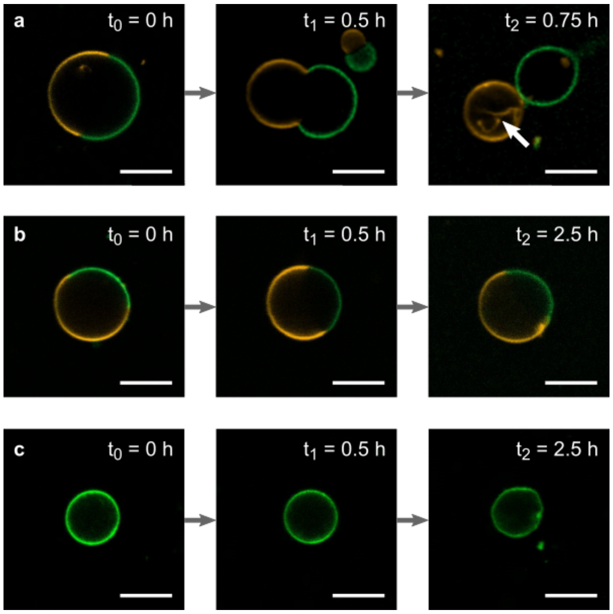

Supporting Figure S3

a Time series of confocal fluorescence images depicting the division process of a phase-separated GUV upon water evaporation. The white arrow highlights the formation of lipid tubes after the division is completed. **b** Control experiment with a phase-separated GUV in a sealed observation chamber preventing water evaporation. **c** Control experiment with a single-phase GUV in an unsealed observation chamber allowing for water evaporation. LissRhod PE labeled the ld phase (orange, $\lambda_{ex} = 561\,\mathrm{nm}$) and 6-FAM-labeled cholesterol-tagged DNA partitioned in the lo phase (green, $\lambda_{ex} = 488\,\mathrm{nm}$). Single-phase GUVs were labeled with Atto488-DOPE (green, $\lambda_{ex} = 488\,\mathrm{nm}$). Scale bars: 10 µm

.

3.4 Figure S4: Phase separation of GUVs produced from different lipid mixtures

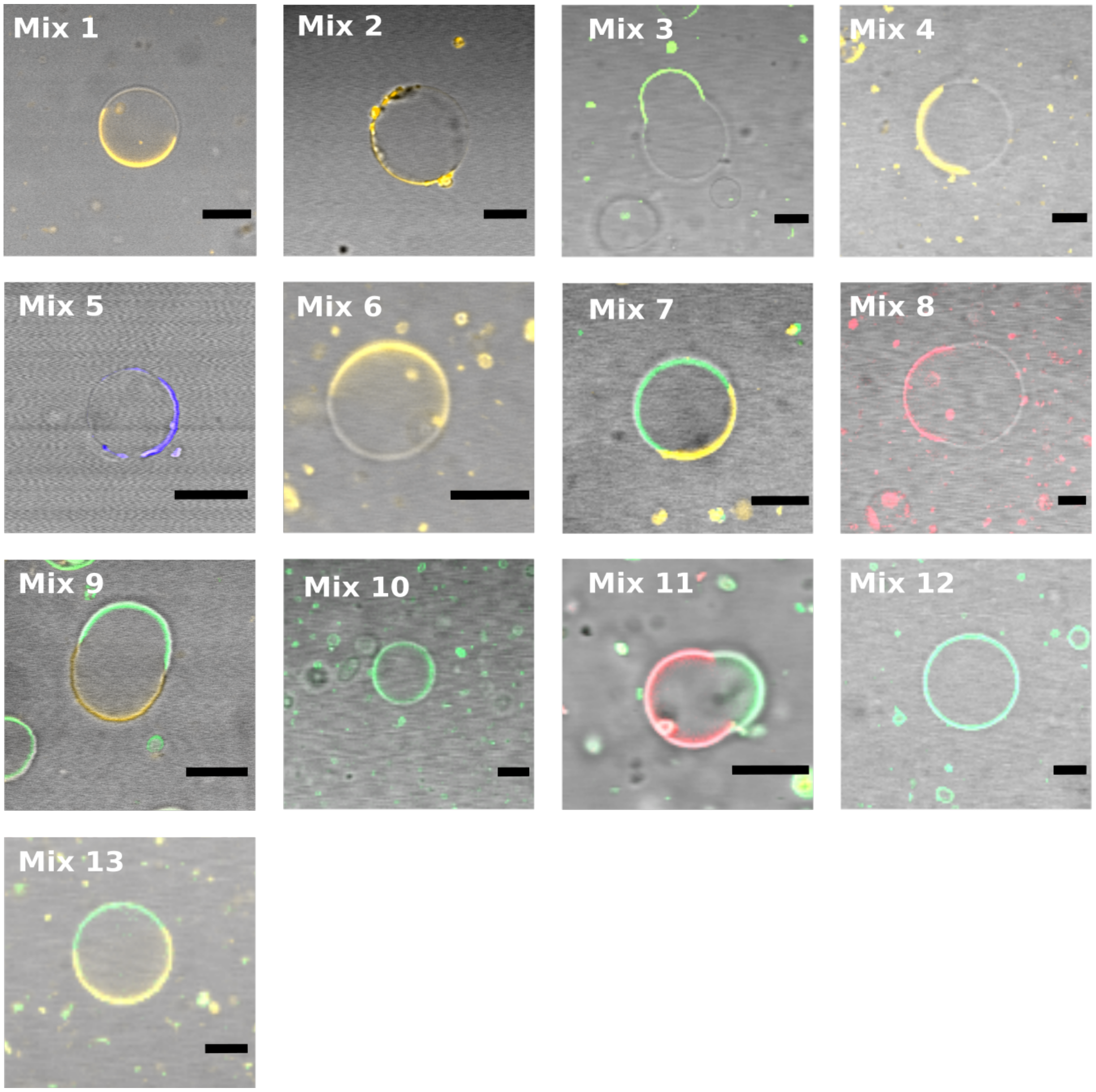

Supporting Figure S4

Representative overlays of confocal fluorescence and brightfield images of GUVs produced from different lipid mixtures as indicated in Tables S2-S4. Cy5 PE was excited with λ_{ex} =633 nm; NBD PE, NBD PC, CF PE, Atto488-DOPE and Chol-6-FAM-DNA were excited with λ_{ex} =488 nm; LissRhod PE was excited with λ_{ex} =561 nm; Chol-Atto390-DNA was excited with λ_{ex} =405 nm. Scale bars: 10 µm.

3.5 Figure S5: Osmolarity mismatch after electroformation

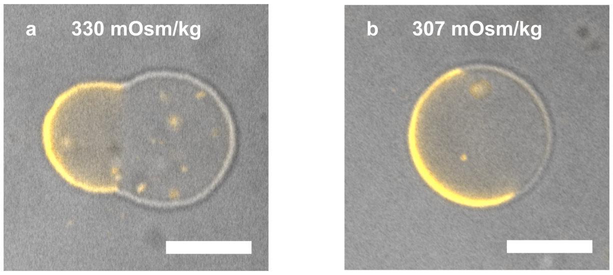

Supporting Figure S5
Overlays of confocal fluorescence (ld phase labeled with LissRhod PE, λ_{ex} =561 nm) and bright field images of GUVs in buffers of different osmolality. (**a**) Directly after electroformation, the GUVs exhibit a non-spherical shape even though the measured osmolality of the vesicle-containing solution does not increase enough for such a significant shape change (325 mOsm kg^{-1} before electroformation, 330 mOsm kg^{-1}). (**b**) If the buffer solution is diluted to 307 mOsm kg^{-1}, the GUV returns to its spherical shape. This may be due to the fact that the vesicles still grow after forming a sealed compartment, leading to a reduced sucrose concentration inside the vesicle. We thus diluted the outer aqueous phase until the GUVs were spherical to achieve our desired initial conditions. Scale bars: 10 µm.

3.6 Figure S6: Necessity of $MgCl_2$ for attachment of cholesterol-tagged DNA

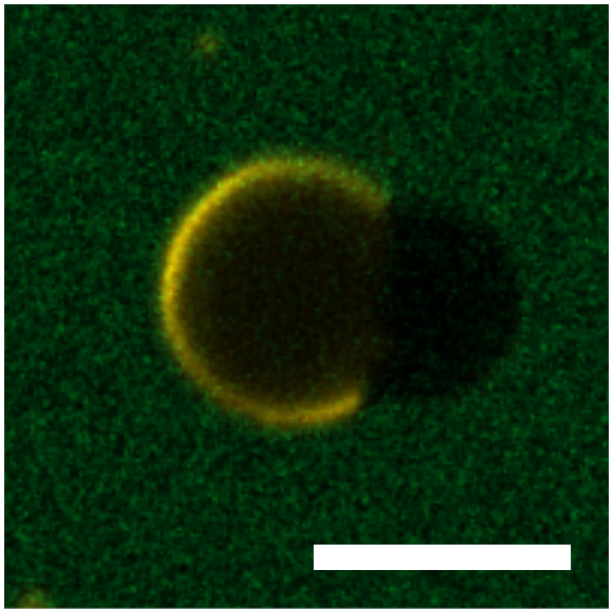

Supporting Figure S6
Confocal fluorescence microscope image of a GUV (ld phase labeled with LissRhod PE λ_{ex} =561 nm, orange) in a solution of 300 mM sucrose, 10 mM HEPES and 1 µM 6-FAM-labeled cholesterol-tagged DNA (λ_{ex} =488 nm, green). Due to lack of $MgCl_2$, the cholesterol-tagged DNA does not attach to the GUV membrane and is homogeneously distributed in the outer aqueous phase instead. For this reason, 10 mM $MgCl_2$ was added to the outer aqueous phase for Figures 1 and 3 (main text), resulting in the attachment of the cholesterol-tagged DNA to the lo phase. Since $MgCl_2$ inhibited the activity of invertase (see Figure S5), it was not possible to label the lo phase in Figure 4 (main text). Scale bar: 10 µm

.

3.7 Figure S7: Reduction of invertase activity in the presence of $MgCl_2$

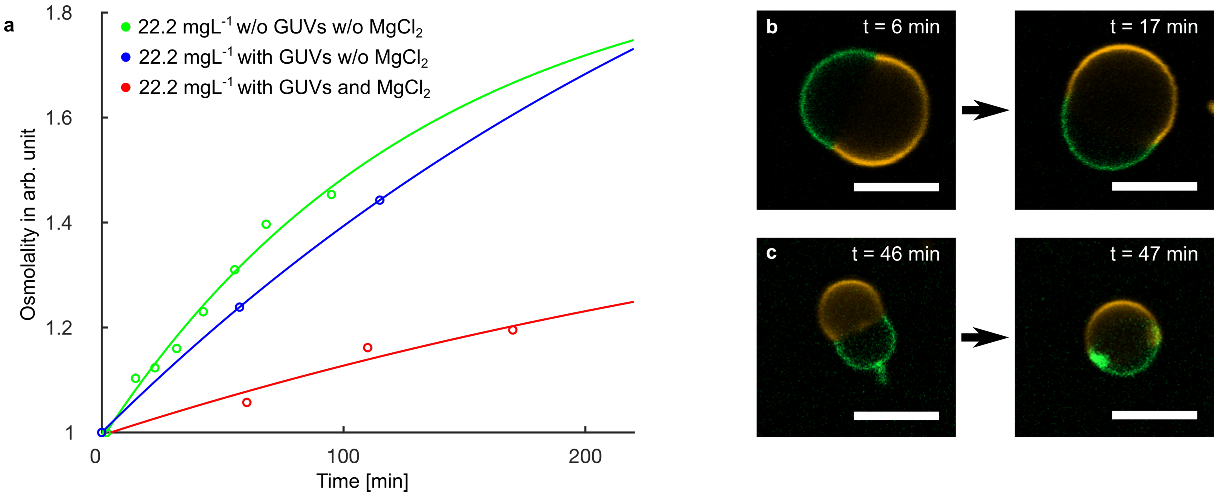

Supporting Figure S7

Effect of $MgCl_2$ on invertase activity in the presence of GUVs. **a** Normalized osmolality measurements over time of a buffer containing 300 mM sucrose, 10 mM HEPES and 22.2 mg l^{-1} in the absence of GUVs and $MgCl_2$ (green); with GUVs but without $MgCl_2$ (blue) and with 10 mM $MgCl_2$ and GUVs (red). Solid lines are limited growth fits. We hypothesize that the reduction of invertase activity in the presence of $MgCl_2$ and GUVs could be caused by a charge-mediated adhesion of the invertase to the GUVs. **b, c** Confocal fluorescence microscopy images of GUVs in the presence of invertase and $MgCl_2$. The ld phase is labeled by LissRhod PE (orange, λ_{ex} =561 nm) and the lo phase is visualized by Chol-6-FAM-DNA (green, λ_{ex} =488 nm). The time after mixing with invertase is indicated. Under these conditions, almost all GUVs did not divide and returned to a spherical shape instead. These observations again point towards an interaction between the invertase and the surface of the GUVs, leading to the reduction of invertase activity. We hence performed the invertase experiments in the absence of $MgCl_2$.

3.8 Figure S8: Gentle shaking overcomes $MgCl_2$-mediated electrostatic interaction between divided GUVs

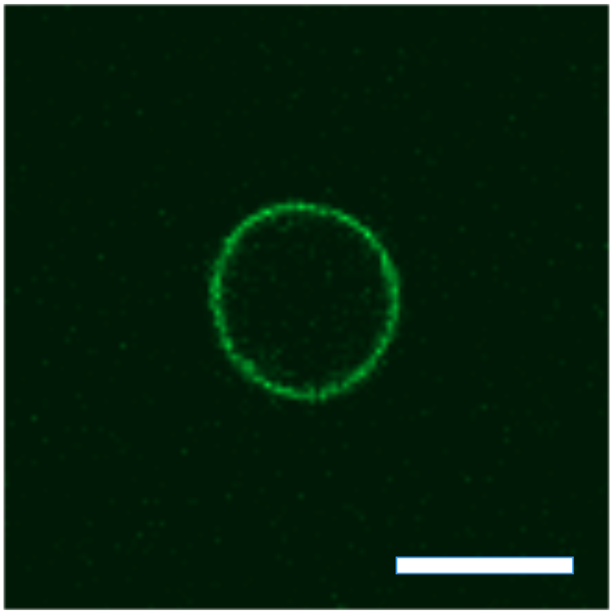

Supporting Figure S8
Confocal fluorescence microscope image of a GUV consisting only of the lo phase (green, 6-FAM labeled cholesterol-tagged DNA partitioned into the lo phase, λ_{ex} =488 nm) after mixing with a higher concentrated sucrose solution leading to an osmolarity ratio of $C/C_0 = 1.44$. At this ratio theoretically all GUVs should be fully divided, yet often adhere to one another if Mg^{2+} is present in the buffer (see Figure 1, main text). Observation of the mixture after gentle shaking, however, yields a high amount of both types of single-phased GUVs. A possible explanation is that Mg^{2+}-mediated electrostatic interactions between divided GUVs could be overcome mechanically due to the mixing process. Scale bar: 10 µm.

3.9 Figure S9: Microfluidic trapping approach

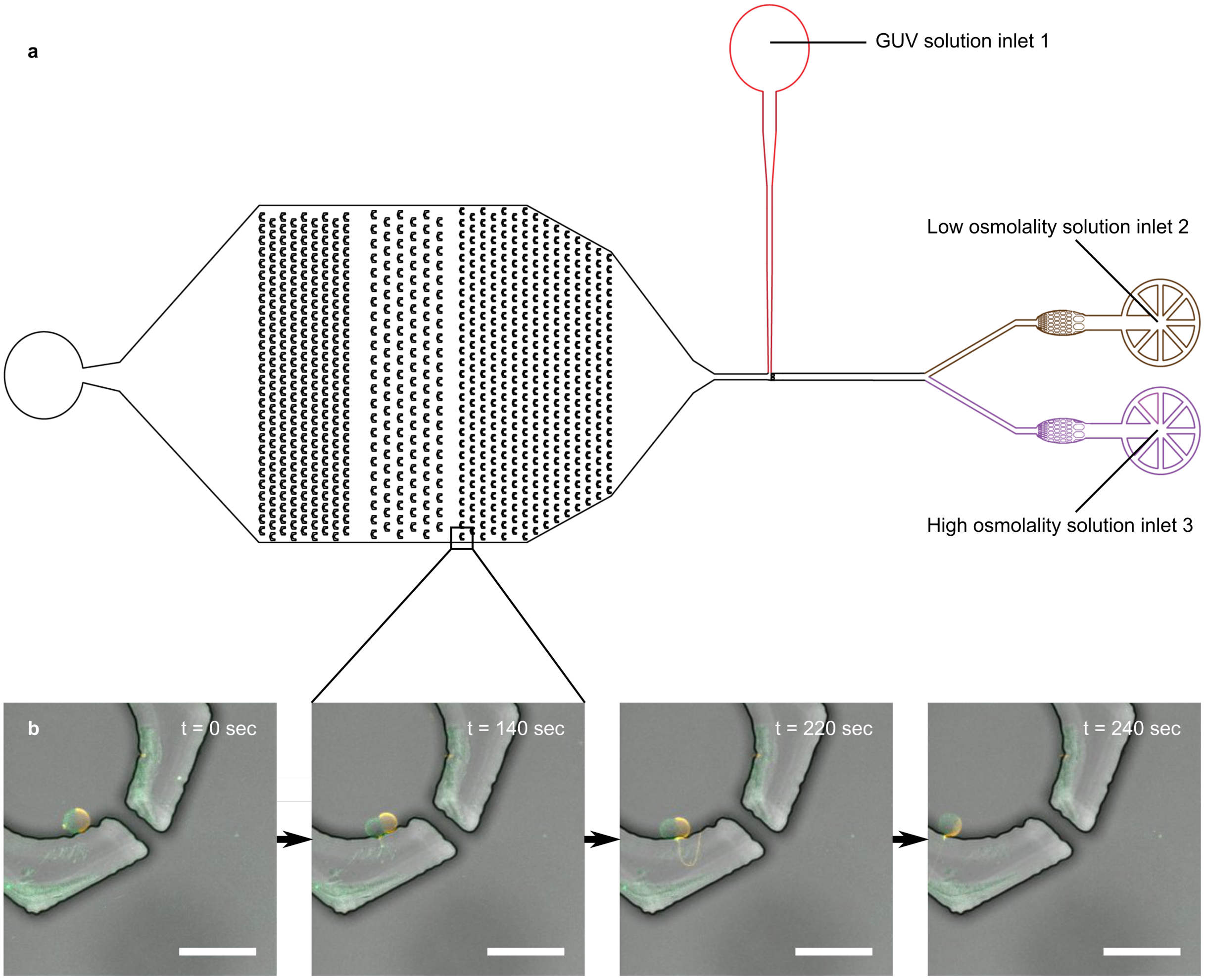

Supporting Figure S9

(Continued on the following page.)

Supporting Figure S9

Microfluidic approach for trapping and observation of GUV division. **a** Sketch of the microfluidic trapping device used for trapping of phase-separated GUVs. First, a solution containing GUVs was flushed into the device via Inlet 1. Subsequently, a low osmolality solution (280 mOsm kg^{-1}, same osmolality as within the GUVs) was flushed into the device at a constant flow rate of 1 µl/ min via Inlet 2. In order to gradually increase the effective osmolality around the GUVs, a second high osmolality solution (600 mOsm kg^{-1}) was flushed in via Inlet 2, starting at a flow rate of 0 µl/ min which was gradually increased to 2 µl/ min over 40 min. **b** Time series of overlays of confocal fluorescence (ld phase labeled with LissRhod PE and lo phase labeled with 6-FAM-labeled cholesterol-tagged DNA, λ_{ex} =561 nm and λ_{ex} =488 nm, respectively) and bright field images of a phase-separated GUV in a trapping device. The interaction of the GUVs with the coverslide and the PDMS microstructures can lead to effects that inhibit the division process. The deformation process can be altered through contact of the GUV with solid interfaces and lead to lipid tubulation rather than splitting as visible in the confocal fluorescence images. Scale bars: 30 µm.

3.10 Figure S10: Characterisation of CMNB-caged fluorescein via absorbance and osmolarity measurements

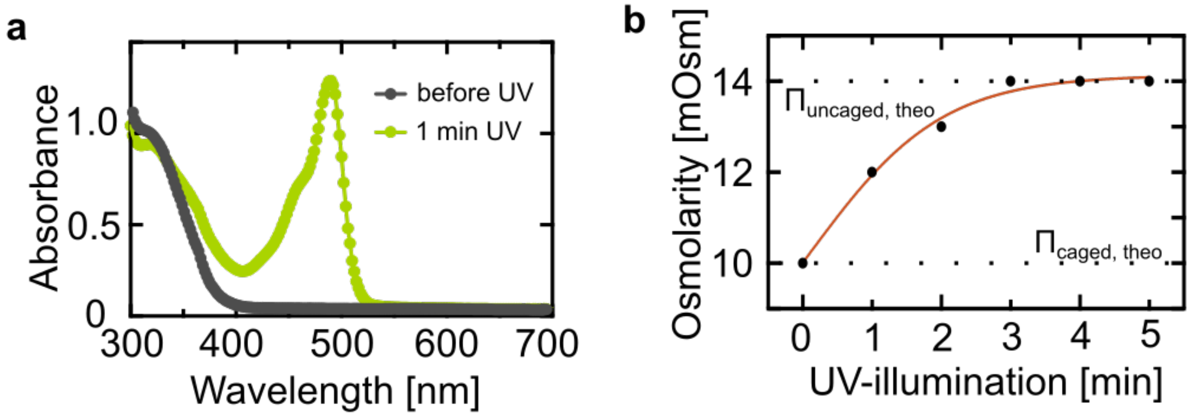

Supporting Figure S10
Characterisation of CMNB-caged fluorescein via absorbance and osmolarity measurements. **a** Absorbance measurements of CMNB-caged fluorescein before and after illumination with a UV-lamp. 2 mM CMNB-caged fluorescein was dissolved in 100 mM Tris and its absorbance was measured from 300-700 nm with a plate reader before (dark green) and after 1 min of UV illumination (light green). The appearance of an absorbance maximum at ≈488 nm after UV-illumination proves successful uncaging of fluorescein. **b** Osmolarity measurements of CMNB-caged fluorescein (final concentration: 2 mM, diluted in MQ) dependent on the UV-illumination time. The maximal theoretical osmolarity of 14 mM – indicating an uncaging efficiency of 100 % (every caged fluorescein splits into 3 parts, see Note S1) – is reached after 3 min. Note that these measurements were performed with a UV-lamp. They cannot directly be correlated with the uncaging dynamics that we obtain with confocal fluorescence microscopy, where we used a 405 nm laser diode for uncaging (see Figure 5, main text and Figure S10).

3.11 Figure S11: Light-triggered division of phase-separated GUVs via uncaging of CMNB-caged fluorescein

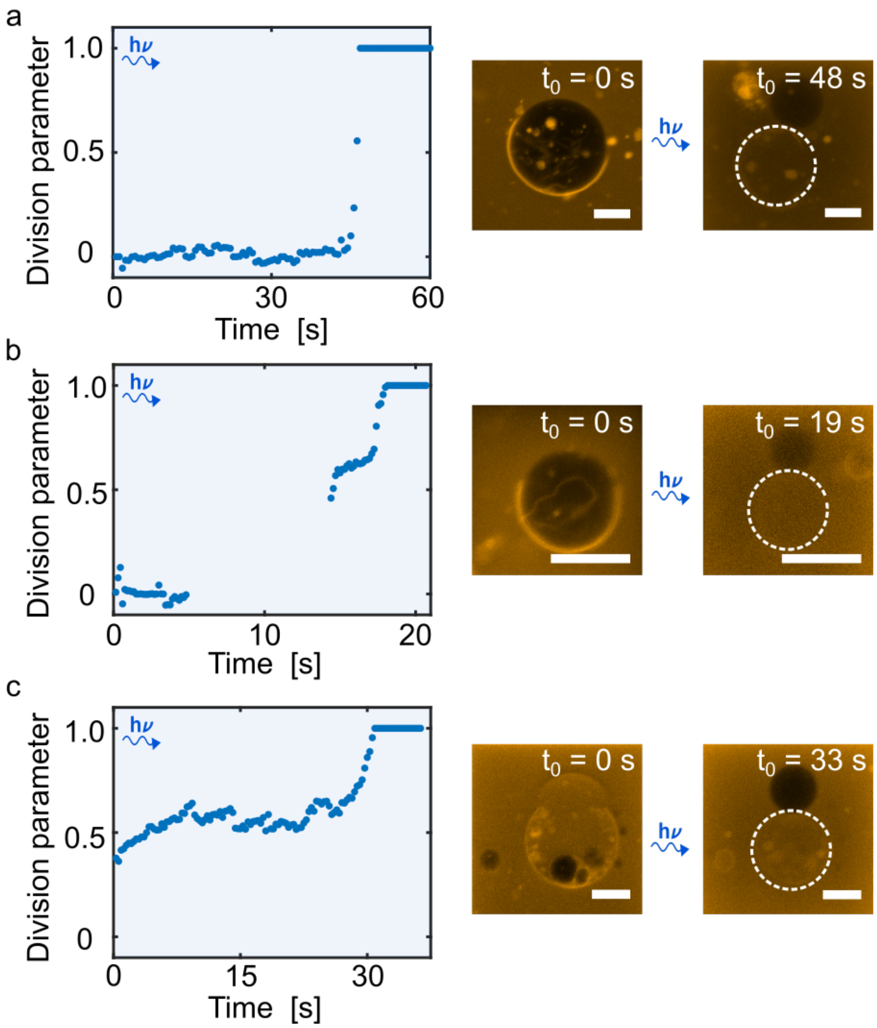

Supporting Figure S11

(Continued on the following page.)

Supporting Figure S11

Light-triggered division of phase-separated GUVs achieved by a local osmolarity increase due to release of CMNB-caged fluorescein. Division parameter as a function of time and corresponding representative confocal fluorescence images of dividing phase-separated GUVs (labelled with LissRhod PE, $\lambda_{ex} = 561\,\text{nm}$). All exemplary traces (**a**-**c**) show that phase-separated GUVs divide within seconds after uncaging of CMNB-caged fluorescein with a 405 nm laser diode. The background fluorescence is caused by bleed-through from the 405 nm excitation. Note that for **b** the shape transformations during the time frames from 5-15 could not be evaluated. The timescales of the light-triggered division process vary from 10 s to 40 s until complete division occurs. This can be explained by two main factors: (i) the differences in the lipid ratio l and (ii) by different uncaging efficiencies depending on the z-position of the GUVs. GUVs with a lipid ratio $l < 0.5$ undergo division at a lower osmolarity ratio and thus at earlier time points during uncaging of CMNB-fluorescein. In addition to this, the absorption and uncaging efficiency depends on the axial position of the GUV within the observation chamber. The further away the GUV is from the bottom cover-slide, the more photons are absorbed by the underlying solution. On top of that, slightly different amounts of BSA on the bottom cover-slide can lead to a less efficient uncaging of CMNB-fluorescein due to absorption of light and therefore a longer timescale until division occurs. We need BSA in order to prevent fusion of the GUVs with the cover slide and imperfections in the coating process cannot be excluded. Scale bars: 10 µm.

3.12 Figure S12: Illumination of CMNB-fluorescein with a 405 nm laser diode leads to uncaging of fluorescein

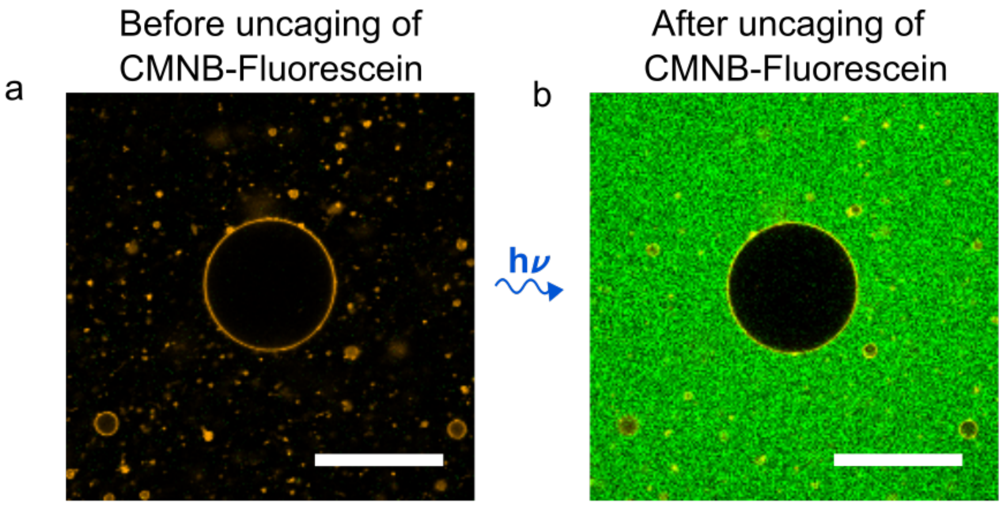

Supporting Figure S12
Illumination of CMNB-caged fluorescein with a 405 nm laser diode leads to uncaging of fluorescein. Confocal fluorescence images of single-phase GUVs (orange, λ_{ex} =561 nm) immersed in CMNB-caged fluorescein (λ_{ex} =488 nm) before (a) and after (b) illumination with a 405 nm diode laser. GUVs (99 % DOPC, 1 % 18:1 Liss Rhod PE) were mixed with an iso-osmolar solution of CMNB-caged fluorescein (final concentration 2 mM) and illuminated with 405 nm for 1 s. This results in uncaging of fluorescein and hence green fluorescence surrounding the GUV. This also indicates the impermeabilty of the caged as well as the uncaged compound across the lipid bilayer. Scale bars: 50 µm.

3.13 Figure S13: 405 nm illumination in absence of CMNB-caged fluorescein does not lead to division of phase-separated GUVs

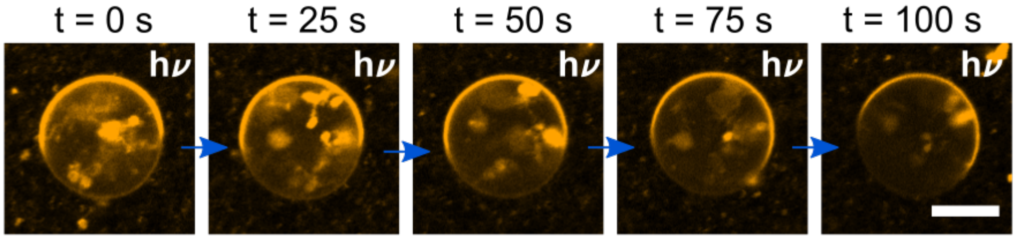

Supporting Figure S13
405 nm illumination in absence of CMNB-caged fluorescein does not lead to division of phase-separated GUVs. Representative confocal fluorescence images of a phase-separated GUV (Table S2, Mix 1, λ_{ex} =561 nm) during illumination with a 405 nm laser diode. The GUV maintains its spherical shape throughout the recorded time frame of 100 s. This proves the need of CMNB-caged fluorescein to locally increase the osmolarity upon 405 nm illumination and hence trigger vesicle division. Scale bar: 10 µm.

3.14 Figure S14: Phase separation can be restored through Ca^{2+}-mediated fusion of single-phased GUVs

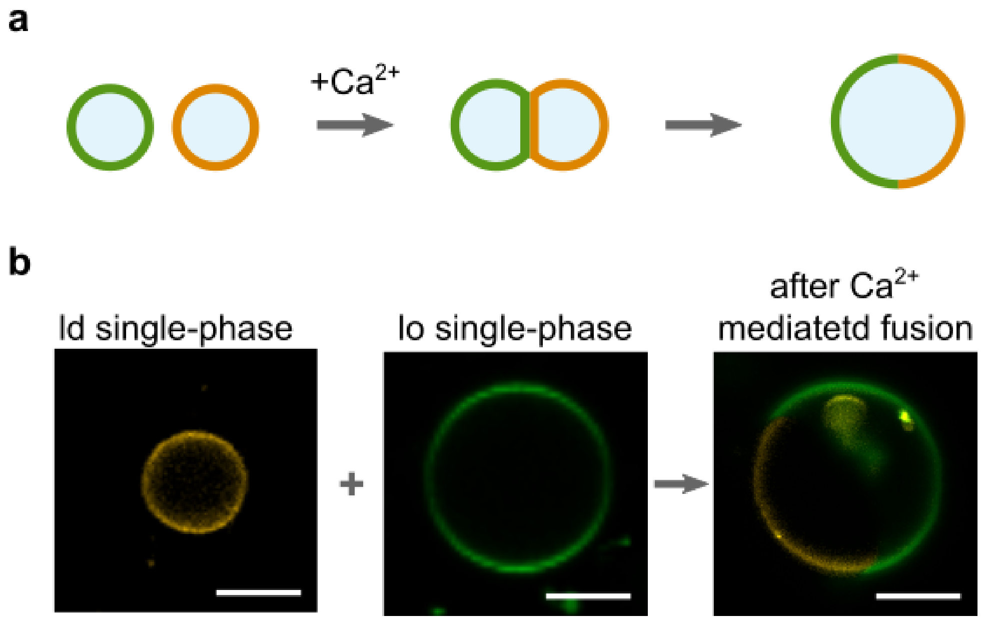

Supporting Figure S14
Ca^{2+}-mediated fusion of a single-phased lo GUV and a single-phased ld GUV. **a** Schematic illustration of formation of phase-separated GUVs via Ca^{2+}-mediated fusion of single-phased GUVs of opposite phases. **b** Confocal fluorescence microscopy images of a single-phased ld (20 % CL, 74.25 % DPPC and 4.75 % DOPC, 1 % Liss Rhod PE λ_{ex} =561 nm, left) and a single-phased lo (49.5 % chol, 49.5 % DOPC, 1 % CF PE, λ_{ex} =488 nm, middle) GUV in 300 mM sucrose. The image on the right hand side shows a phase-separated GUV after mixing the lo GUVs with the ld GUVs in the presence of 20 mM $CaCl_2$. The presence of both fluorescent dyes in the same vesicle and the phase separation can only be explained by fusion of a lo GUV with a ld GUV. A fusion event leads to an overall lipid mixture that exhibits phase separation and contains both fluorescent dyes. Note that the lipid mixture after fusion represents the mixture used for the division experiments in the main. Scale bar: 10 µm.

3.15 Figure S15: Phase separation can be restored through Ca^{2+}-mediated fusion of SUVs to single-phased GUVs

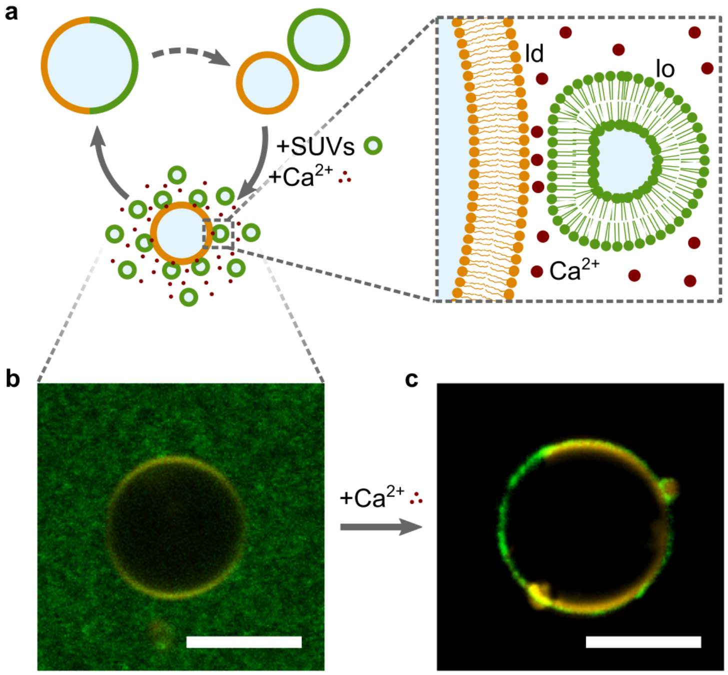

Supporting Figure S15
Phase separation can be restored through Ca^{2+}-mediated fusion of SUVs to single-phased GUVs. **a** Schematic illustration of a sustainable vesicle growth and division cycle mediated by calcium ions. **b, c** Representative confocal fluorescence images of fluorescently-labeled ld-phase GUVs (orange, λ_{ex} =561 nm) in a feeding bath of lo-phase SUVs (green, λ_{ex} =488 nm) prior (**b**) and after (**c**) addition of 20 mM $CaCl_2$. SUV fusion restores the phase separation of the GUV.

3.16 Figure S16: Tocopherol-tagged DNA is homogeneously distributed in the lo- and ld-phases of phase-separated GUVs

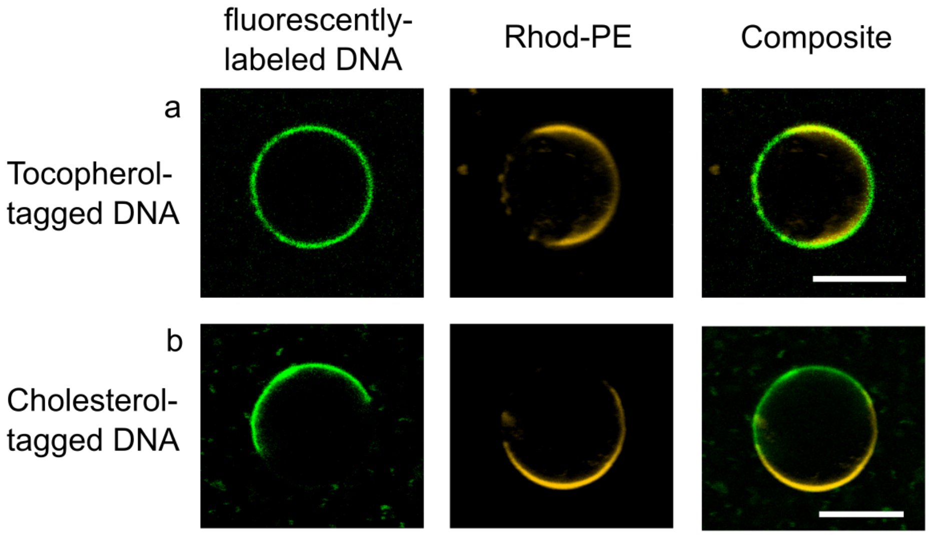

Supporting Figure S16
Tocopherol-tagged DNA is homogeneously distributed in the lo- and ld-phases of phase-separated GUVs. Confocal fluorescence images of GUVs (Lipid Mix 1) upon addition of fluorescently-labeled DNA (λ_{ex} =405 nm) with (a) a tocopherol and (b) a cholesterol modification (left images). The ld phase is labelled with 18:1 Liss Rhod PE (λ_{ex} =561 nm, middle images). Composite images are shown on the right. Whereas cholesterol-tagged DNA is preferentially localized in the lo-phase of phase-separated GUVs, tocopherol-tagged DNA is homogeneously distributed within both lo- and ld-phases. For the DNA fusion experiments (Figure 6, main text) we can thus use cholesterol-tagged DNA to selectively label the lo-phase and a complementary tocopherol-tagged DNA for the ld phase. Here, 1 µM fluorescently-labeled (Atto390) DNA, 10 mM $MgCl_2$ and 1x PBS matching the osmolarity of the sucrose solution used for electroformation were added to a solution of phase-separated vesicles. Scale bar: 10 µm.

3.17 Figure S17: DLS of lo SUVs

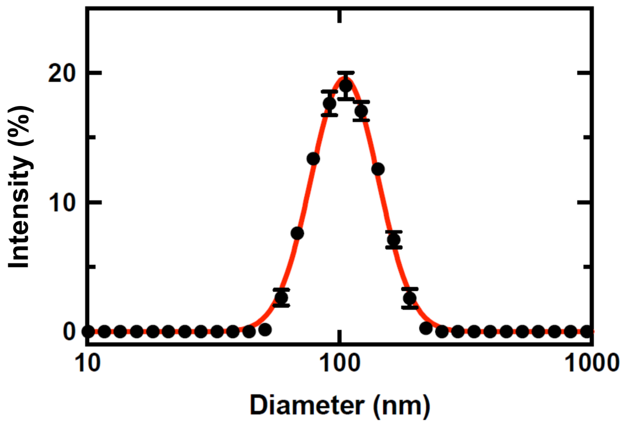

Supporting Figure S17
Intensity distribution of the diameters of lo SUVs used for the regrowth of phase-separated GUVs, determined by dynamic light scattering (DLS). 3 mM SUVs (49.5 % DOPC, 49.5 % cholesterol, 1 % CF PE) were produced using the extrusion method with a 100 nm membrane filter in a 300 mM sucrose solution. The measurements were performed using a solution of 1 mL of 100 µM SUVs. The average and standard deviation values were determined from 10 individual runs. The distribution was fitted with a log-normal Gaussian distribution (red line), revealing a mean diameter of 115.1 ± 1.4 nm. This slightly larger measured hydrodynamic diameter is typical for SUVs extruded with a 100 nm membrane.

3.18 Figure S18: DNA-mediated vesicle fusion leads to growth of lo-phase of initially single-phased GUVs

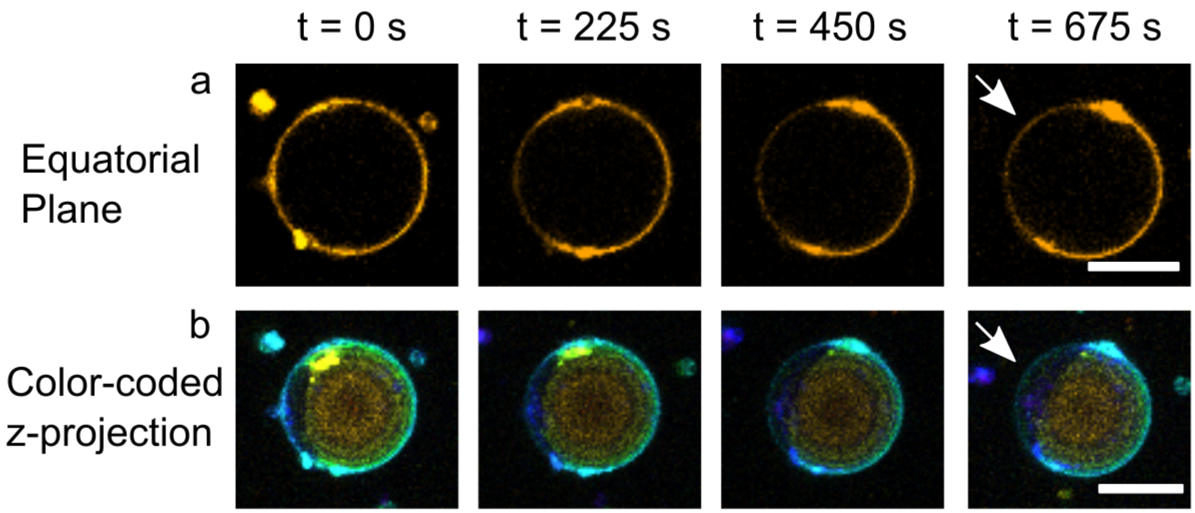

Supporting Figure S18

DNA-mediated vesicle fusion leads to growth of lo-phase of initially single-phased ld GUVs. Confocal fluorescence time series of the equatorial plane (a) as well as color-coded z-projection (b) of a growing fluorescently-labeled GUV (18:1 Liss Rhod PE, λ_{ex} =561 nm) in presence of SUVs. Lo-phase SUVs (50 % cholesterol, 5 0% DOPC) and ld-GUVs (20 % CL, 74.25 % DPPC, 4.75 % DOPC, 1 % Liss Rhod PE) were incubated with complementary cholesterol-tagged and tocopherol-tagged DNA, respectively. After incubation, lo-SUVs and ld-GUVs were mixed in presence of 10 mM $MgCl_2$ and imaged over time. Duplex formation brings SUVs and GUVs in close proximity, eventually leading to vesicle fusion. This can be seen in form of the growing lo-phase of the GUV. Scale bar: 20 µm.

3.19 Figure S19: DNA-functionalized lo SUVs do not fuse to plain ld GUV

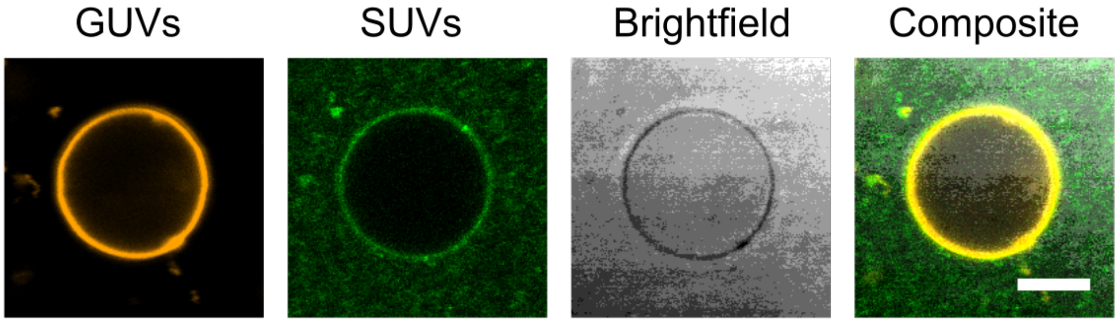

Supporting Figure S19
DNA-functionalized lo SUVs do not fuse to bare ld GUV. Confocal fluorescence images of a fluorescently-labeled single-phase GUV (18:1 Liss Rhod PE, λ_{ex} =561 nm) in presence of SUVs incubated with cholesterol-tagged fusion DNA as well as cholesterol-tagged 6-FAM DNA (for labelling purposes, λ_{ex} =488 nm, see Methods). Lo-phase SUVs (50 % cholesterol, 50 % DOPC) were incubated with 1 µM DNA #1 and #2 in presence of 5 mM $MgCl_2$ for 20 minutes and afterwards mixed with ld-GUVs (20 % CL, 74.25 % DPPC and 4.75 % DOPC, 1 % Liss Rhod PE). Note that the tocopherol-tagged fusion DNA #3 was omitted. In this control experiment, no vesicle fusion leading to the formation of phase-separated GUVs could be detected. However, the presence of magnesium ions leads to the attachment of SUVs to the GUVs as visible in the representative confocal fluorescence image. It is conceivable that the single-stranded DNA acts as a spacer which prevents fusion. Scale bar: 10 µm.

4 Supporting Notes

4.1 Note S1: Derivation of theoretical prediction

If we look at the initially spherical vesicle with an initial raidus of r_0 and a lipid ratio l the initial volume and areas are given by

$$V_0 = \frac{4}{3} \cdot \pi \cdot r_0^3 \tag{1}$$
$$A_{tot} = 4 \cdot \pi \cdot r_0^2 \tag{2}$$
$$A_{ld} = l \cdot A_{tot} \tag{3}$$
$$A_{lo} = (1 - l) \cdot A_{tot}. \tag{4}$$

The lipid phases can be represented as sphere segments with radius r_0 and a height of

$$h_{ld} = A_{ld}/(2 \cdot \pi \cdot r_0) = 2 \cdot l \cdot r_0 \tag{5}$$
$$h_{lo} = A_{lo}/(2 \cdot \pi \cdot r_0) = 2 \cdot (1 - l) \cdot r_0 \tag{6}$$

and a base area of

$$s_0 = \sqrt{\frac{A_{ld}}{\pi} - h_{ld}^2} = 2 \cdot r_0 \cdot \sqrt{l - l^2}. \tag{7}$$

If the outer osmolarity is increased from C_0 to C the vesicle will deflate and its volume reduce to

$$V = V_0 \cdot \frac{C_0}{C}. \tag{8}$$

We assume that the vesicle will deform in a way that the line tension, i.e. the circumference of the base of the spherical caps, gets minimized. This leads again to two spherical caps for each phase with the same reduced radius of the base of the spherical cap s. We can now define a division parameter d that quantifies the progress of the division process as

$$d = 1 - s/s_0. \tag{9}$$

The volume of each cap is then given by

$$V_i = \frac{\pi}{3} \left(r_i \pm \sqrt{r_i^2 - s^2} \right) \left(s^2 + r_i \left(r_i \pm \sqrt{r_i^2 - s^2} \right) \right) \tag{10}$$

with $i = ld, lo$ and r_i the respective radius of the sphere segments. The area of the new sphere segments stay the same as before and are given by

$$A_i = 2 \cdot \pi \cdot r_i \cdot \left(r_i \pm \sqrt{r_i^2 - s^2} \right) \tag{11}$$

Inserting Eq. (11) into Eq. (10) leads to

$$V_i = \frac{\pi}{3} \frac{A_i}{2\pi r_i} \left(s^2 + \frac{A_i}{2\pi} \right) \tag{12}$$

The radii r_i of the spherical caps are given by

$$r_i = \frac{A_i}{2\pi h_{i,2}} \tag{13}$$

with

$$h_{i,2} = \sqrt{\frac{A_i}{\pi} - s^2} \tag{14}$$

the respective new height of the sphere segments. Inserting Eqs. (7), (9), (13) and (14) into Eq. (12) leads to

$$V_i = \frac{\pi}{3} \sqrt{\frac{A_i}{\pi} - 4r_0^2(l - l^2)(1-d)^2} \left(4r_0^2(l - l^2)(1-d)^2 + \frac{A_i}{2\pi} \right) \tag{15}$$

By replacing A_i with Eq. (3) and Eq. (4) respectively and using Eq. (1) we obtain

$$V_{ld} = V_0 \sqrt{l - (1-d)^2 \cdot (l - l^2)} \cdot \left(2 \cdot (1-d)^2 (l - l^2) + l \right) \equiv V_0 \cdot T_1 \tag{16}$$

$$V_{lo} = V_0 \sqrt{((1-l) - (1-d)^2 \cdot (l - l^2)} \cdot \left(2 \cdot (1-d)^2 (l - l^2) + (1-l) \right) \equiv V_0 \cdot T_2. \tag{17}$$

Using Eq. (8) leads to the the direct dependence of the osmolarity ratio C/C_0 and the division parameter d

$$\frac{C}{C_0} = \frac{V_0}{V} = \frac{V_0}{V_{ld} + V_{lo}} = \frac{1}{T_1 + T_2}. \tag{18}$$

For symmetric vesicles with $l = 0.5$ Eq. (18) becomes

$$C/C_0 = \frac{2}{\sqrt{2 - (1-d)^2}((1-d)^2 + 1)}. \tag{19}$$

4.2 Note S2: Considerations for osmolarity matching with CMNB-caged fluorescein

CMNB-caged fluorescein was dissolved in 100 mM Tris buffer to a final concentration of 50 mM. Since CMNB-caged fluorescein is twice negatively charged, the solution furthermore contains 100 mM potassium ions. This yields a total numerical osmolarity

of 250 mOsm. For the osmolarity measurements (Supporting Figure S9**b**), the caged fluorescein-containg solution was diluted in MQ to a final concentration of 2 mM CMNB-caged fluorescein leading to an overall osmolarity of 10 mOsm ($\Pi_{caged,theo}$). Thus, an uncaging efficieny of 100 % leads to a rise of the osmolarity from 10 mOsm to 14 mOsm ($\Pi_{uncaged,theo}$) since each uncaged fluorescein molecule is accompanied by two CMNB molecules.
For the light-mediated division experiments, GUVs were electroformed in a solution of 13 mM sucrose and mixed with an isoosmolar solution of CMNB-caged fluorescein in a ratio of 1:2. Therefore, the final CMNB-caged fluorescein concentration is 1.73 mM, which, in a closed system, would yield a maximum osmolarity ratio of 1.27 assuming an uncaging efficiency of 100 % (as obtained in the osmolarity measurements in Figure S9**a**). However, local concentration inhomogenities, pipetting errors and different diffusion speeds of the uncaged molecules can lead to even higher local osmolarity changes. Since the lipid ratio for the vesicle presented in Figure 5 is $l = 0.63$, it should divide at an osmolarity ratio of 1.37 according to our model. This is hence in good agreement with the expected change in osmolarity due to the uncaging of CMNB-fluorescein.

4.3 Note S3: Osmolarity vs. osmolality

It is important to note that the measurements produced by the Osmomat 030 (Genotec GmbH) indicate the osmolality b of the sample solution. However, according to van't Hoffs law the osmotic pressure depends on the total particle concentration in the solution (osmolarity) C: $\Pi = CRT$, where C is the osmolarity of the solution, R the ideal gas constant and T the temperature. The osmolarity depends linearly on the osmolality $C = (\rho_S - c_a) \cdot b$ where ρ_S is the density of the solution and c_a is the anhydrous solute concentration. The prefactor $(\rho_S - c_a)$ is dependent on the solute. It can be neglected in most cases since $(\rho_S - c_a) \approx 1\,\mathrm{kg\,l^{-1}}$ [9]. However, for high concentrations of sugars as have been used here, the prefactor can deviate from $1\,\mathrm{kg\,l^{-1}}$. Since we look at the osmotic pressure ratio C/C_0, the effect cancels out nevertheless. It can only play a role when using different solutions for the inner and outer phase like in Figure 3 (main text). However, even then, the deviation of the osmotic pressure ratio is negligible: For the solutions that were used here, $(\rho_S - c_a)$ did not deviate more than 15% from $1\,\mathrm{kg\,l^{-1}}$. Hence, the deviation of the osmotic pressure ratio C/C_0 is below 1%. Furthermore, Moser and Frazer suggested the that the osmotic pressure is better described by $\Pi = \frac{n}{V'}RT = \rho_\mathrm{w} bRT$, for higher concentrations of glucose or sugar with V' the volume of pure water in the solution, ρ_w is the density of pure water and b is the osmolality of the solution [10]. Therefore, we used the osmolality instead of the osmolarity for the calculations.

5 Supporting Videos

5.1 Video S1: Conceptual model and confocal fluorescence time lapse of GUV division

Video S1 shows the theoretical prediction as well as experimental observations of the division process. It first visualizes the deformation and division process for increasing osmolarity ratios based on the predictions of the theoretical model. It then shows two independent time series of confocal fluorescence images of the division process of a phase-separated GUV (27.125 % DOPC, 24,75 % cholesterol, 37.125 % DPPC, 10 % CL, 1 % LissRhod PE) in an unsealed observation chamber which allows for water evaporation. The ld phase is labeled with LissRhod PE (λ_{ex} =561 nm) and 6-FAM-labeled cholesterol-tagged DNA (λ_{ex} =488 nm) partitioned into the lo phase. The time series were taken by manually tracking free-floating GUVs using the x-y-z piezo stage of the confocal microscope. The videos were generated by tracking the position of the GUV within the confocal image using the TrackMate plugin (ImageJ). Images were then truncated accordingly and the brightness- and contrast-adjusted fluorescence channels were overlayed. Note that within the confocal time series, other free-floating GUVs appear in the field of view, which show similar deformation stages as the GUV that was tracked.

5.2 Video S2: Division of phase-separated GUV triggered by enzymatic decomposition

Video S2 shows an overlay of confocal fluorescence and brightfield time series depicting the division process of a phase-separated GUV, whereby the osmolarity increase is achieved by the enzymatic decomposition of sucrose. Note that the phase-separated GUV is composed of a distinctly different lipid mixture (33 % SM, 33 % cholesterol and 33 % DOPC labeled with 1 % NBD PC λ_{ex} =488 nm, see Table S4, Mix No. 3). The fact that we still observe full division including neck fission indicates that our division mechanism is robust with respect to lipid type. GUVs were prepared via the electroformation method in 300 mM sucrose. After mixing with invertase, resulting in a final concentration of 44 mg l^{-1} invertase the GUVs were observed in a sealed observation chamber under a confocal fluorescence microscope. NBD PC labeled the ld phase. The video was generated by tracking the position of the GUV within the confocal image using the TrackMate plugin (ImageJ). Images were then truncated accordingly and the brightness- and contrast-adjusted fluorescent and brightfield channel were overlayed. Scale bar: 10 µm.

5.3 Video S3: Light-triggered division of phase-separated GUVs by uncaging of CMNB-fluorescein

Video S3 shows a confocal fluorescence time series of a phase-separated GUV (Supporting Table S4, Mix 1) before and during illumination with a 405 nm laser diode. The time series before 405 nm illumination depicts brightfield and fluorescence composite images of a phase-separated GUV (λ_{ex} =561 nm) for two minutes. The GUV does not show any deformation of its spherical shape. During 405 nm illumination, the GUV deforms at the phase-boundary leading to vesicle division within seven seconds due to uncaging of CMNB-fluorescein and hence a local osmolarity increase (see also Figure 5**e** in the main text for the calculation of the division parameter). After division, another composite confocal fluorescence image shows the divided vesicles post 405 nm illumination. Light-triggered release of caged fluorescein offers full spatiotemporal control of the division process of the selected vesicle.

5.4 Supporting Video S4: Calcium-mediated fusion of single-phase GUVs to restore phase-separation

Composite confocal fluorescence time series of single-phase GUVs (labeled with either Atto488-DOPE λ_{ex}=488 nm or Liss Rhod-PE λ_{ex}=561 nm) in an observation chamber to which 10 mM $CaCl_2$ were added. Calcium-ions lead to an attachment and clustering of GUVs eventually resulting in vesicle fusion within minutes. This shows the possibility to regrow phase-separated GUVs.

References

[1] Angelova, M. I. & Dimitrov, D. S. Liposome electroformation. *Faraday Discussions of the Chemical Society* **81**, 303 (1986).

[2] Phan, M. D. & Shin, K. Effects of cardiolipin on membrane morphology: a langmuir monolayer study. *Biophysical journal* **108**, 1977–1986 (2015). URL `https://pubmed.ncbi.nlm.nih.gov/25902437`.

[3] Mills, T. T., Huang, J., Feigenson, G. W. & Nagle, J. F. Effects of cholesterol and unsaturated dopc lipid on chain packing of saturated gel-phase dppc bilayers. *General physiology and biophysics* **28**, 126–139 (2009). URL `https://pubmed.ncbi.nlm.nih.gov/19592709`.

[4] Beales, P. A., Nam, J. & Vanderlick, T. K. Specific adhesion between dna-functionalized “janus” vesicles: size-limited clusters. *Soft Matter* **7**, 1747–1755 (2011).

[5] Suga, K. & Umakoshi, H. Detection of nanosized ordered domains in dopc/dppc and dopc/ch binary lipid mixture systems of large unilamellar vesicles using a tempo quenching method. *Langmuir* **29**, 4830–4838 (2013). URL https://doi.org/10.1021/la304768f.

[6] Kahya, N., Scherfeld, D., Bacia, K., Poolman, B. & Schwille, P. Probing lipid mobility of raft-exhibiting model membranes by fluorescence correlation spectroscopy. *Journal of Biological Chemistry* **278**, 28109–28115 (2003). URL http://www.jbc.org/content/278/30/28109.abstract. http://www.jbc.org/content/278/30/28109.full.pdf+html.

[7] Chan, Y.-H. M., van Lengerich, B. & Boxer, S. G. Effects of linker sequences on vesicle fusion mediated by lipid-anchored dna oligonucleotides. *Proceedings of the National Academy of Sciences* **106**, 979–984 (2009).

[8] Rodriguez, N., Pincet, F. & Cribier, S. Giant vesicles formed by gentle hydration and electroformation: A comparison by fluorescence microscopy. *Colloids and Surfaces B: Biointerfaces* **42**, 125 – 130 (2005). URL http://www.sciencedirect.com/science/article/pii/S0927776505000603.

[9] S̃klubalová, Z. & Zatloukal, Z. Conversion between osmolality and osmolarity of infusion solutions. *Scientia Pharmaceutica* **77**, 817–826 (2009).

[10] Morse, H. N. & Frazer, J. C. W. The osmotic pressure and freezing-points of soiutions of cane-sugar. *American Chemical Journal (Baltimore)* **34**, 1–99 (1905).
